# Combustion versus Gasification in Power- and Biomass-to-X Processes: An Exergetic Analysis

**DOI:** 10.1021/acsomega.4c05549

**Published:** 2024-11-21

**Authors:** Simone Mucci, Alexander Mitsos, Dominik Bongartz

**Affiliations:** † Process Systems Engineering (AVT.SVT), RWTH Aachen University, 52074 Aachen, Germany; ‡ Department of Chemical Engineering, KU Leuven, 3001 Leuven, Belgium; § JARA-ENERGY, 52056 Aachen, Germany; ∥ Energy Systems Engineering (ICE-1), Forschungszentrum Jülich, 52425 Jülich, Germany; ⊥ EnergyVille, 3600 Genk, Belgium

## Abstract

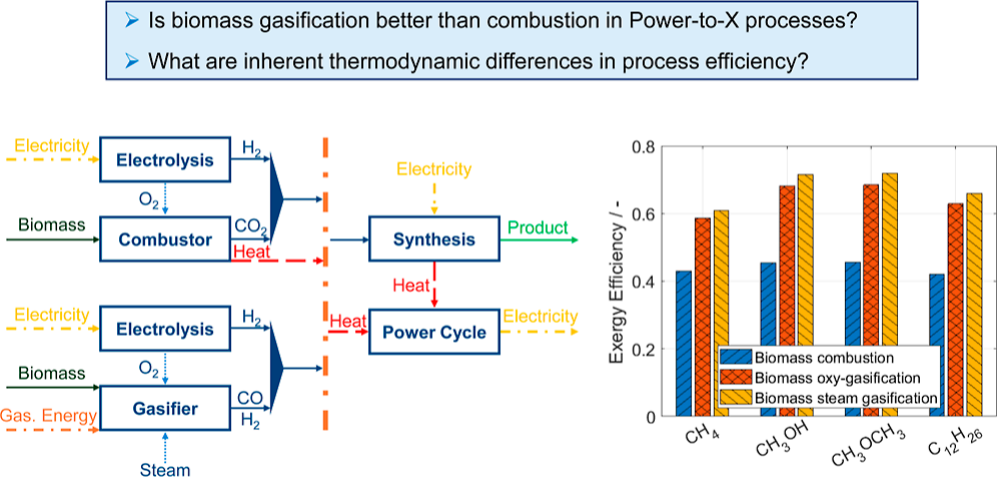

Residual biomass is a promising carbon feedstock for the production of electricity-based organic chemicals and fuels since, unlike carbon dioxide captured from point sources or air, it also has a valuable energy input. Biomass can be converted into an intermediate stream suitable for Power-to-X processes mainly via combustion or gasification. Such combined processes are generally called biohybrid or Power- and Biomass-to-X processes. To investigate the potential of biomass utilization in Power- and Biomass-to-X processes and identify inherent efficiency differences between these pathways, we model the process units with simple mass and energy balances considering empirical parameters for the key process units and perform an exergetic analysis. The analysis is conducted for several molecules of interest for the chemical and transport sectors with different C:H:O ratios, i.e., methane, methanol, dimethyl ether, and dodecane. For all considered products, the Power- and Biomass-to-X processes with biomass gasification, either with pure oxygen or steam as oxidizing agents, have a significantly higher (∼15–20 percentage points) exergy efficiency. This difference is mainly due to the lower exergy loss for water electrolysis since a lower amount of hydrogen is needed and to the higher exergy efficiency of the gasification unit compared to that of the combustion unit. Therefore, gasification-based Power- and Biomass-to-X processes have clear thermodynamic advantages in the ideal case. These conclusions obtained with the simple models are confirmed by modeling a Power- and Biomass-to-Methanol process in detail, also accounting for practical factors such as side reactions, incomplete reactant conversion, and ash formation.

## 1 Introduction

The synthesis of products using green electricity (Power-to-X) can contribute to defossilizing the chemical and transport sectors. In addition to electricity, the production of organic chemicals and fuels with a low carbon footprint requires a sustainable carbon source. Carbon dioxide captured from point sources, e.g., cement and steel plants, or directly from air^1^ is often considered as feedstock. Another promising carbon feedstock is biomass, which, unlike carbon dioxide, also has a valuable energy content.

There are two biomass conversion pathways, i.e., biochemical and thermochemical, for the production of chemicals, and both have advantages and disadvantages depending on the biomass feedstock and the target product. Moreover, the thermochemical conversion of biomass can represent a viable alternative or a complement to biochemical conversion. For instance, some biomass fractions can be directly and rather easily used for the production of valuable products, e.g., fermentable sugars, levulinic acid,^2^ and cellulose nanofibers,^3^ thus exploiting the existing functionalized molecular structures,^4^ while the residual fractions, e.g., lignin, require more intense conversion processes, e.g., thermochemical conversion processes.^5^

Several thermochemical conversion processes of biomass, e.g., pyrolysis, have been proposed, but combustion and gasification processes are the most mature.^6,7^ Combustion and gasification processes can convert almost any biogenic feedstock into some base intermediates, e.g., CO_2_, CO, and H_2_, which can then be used to synthesize the chemicals of interest. These intermediates can be combined with Power-to-X products such as green hydrogen. This way, residual biomass can be used as carbon feedstock in Power-to-X processes; the combination is termed “Power- and Biomass-to-X” or “biohybrid” process^8^ and can have synergetic effects. For ethanol production, it was shown that the combination results in a better trade-off of economic and environmental objectives than either purely electrical or purely biogenic production.^8^ Techno-economic trade-offs have also been shown for Power- and Biomass-to-Liquid.^9−11^

Many works can be found in the literature about these thermochemical conversion technologies, especially gasification,^6,12−14^ and their modeling.^7,15^ For instance, different types of gasification processes have been compared concerning energy and exergy efficiency.^15,16^ Also, gasification units have been considered in Biomass-to-X processes.^17,18^ Combustion-based and gasification-based Power- and Biomass-to-X processes have been compared for kerosene production^19^ but without shedding light on the fundamental thermodynamic reasons for the difference in kerosene production efficiency. Furthermore, a systematic comparison of the main thermochemical biomass conversion processes, i.e., combustion and gasification, in the Power- and Biomass-to-X context that also analyzes their fundamental differences in the process efficiency is still missing. Although it is expected that combustion-based Power- and Biomass-to-X processes are less efficient than gasification-based processes since the carbon atoms are first fully oxidized and then reduced, they offer practical advantages, e.g., in flue gas cleaning. Therefore, we want to quantify how big the efficiency differences in combustion-based and gasification-based Power- and Biomass-to-X processes are when considering empirical parameters for the key process units and identify the underlying thermodynamic reasons for such efficiency differences between these biomass conversion pathways.

To address these aspects, we model Power- and Biomass-to-X processes composed of a biomass conversion unit based on either combustion or gasification, a low-temperature electrolyzer, and a product synthesis unit. Additionally, a power cycle unit is considered to convert heat from the exothermic units into electricity to reduce the net electricity demand of the Power- and Biomass-to-X process. The units are modeled with mass and energy balances; we intentionally keep the models simple and general, e.g., not dependent on reactor configurations, operating conditions, and catalyst performance, which might change due to technological improvements, in order to identify inherent efficiency differences between the considered Power- and Biomass-to-X processes and highlight their potential and limitations. Thus, the process efficiencies calculated with these simple and general models represent a benchmark of the efficiency that Power- and Biomass-to-X processes modeled in more detail can potentially achieve.

An exergy analysis is then performed to compare the two pathways, i.e., combustion-based and gasification-based Power- and Biomass-to-X processes, by considering several target products with different C:H:O ratios, i.e., methane, methanol, dimethyl ether, and dodecane. Furthermore, a sensitivity analysis concerning the electrolyzer and power cycle efficiencies and the carbon feedstock composition is carried out to evaluate the influence of the key process parameters. Finally, practical aspects that can affect the efficiency beyond the fundamental thermodynamic effects as well as the cost are discussed. Moreover, we compared the two pathways for a Power- and Biomass-to-Methanol process modeled in detail to verify the results obtained with the simpler models.

The remainder of the paper is structured as follows: in Sections 2 and 3, the investigated Power- and Biomass-to-X processes and their models are described, respectively. Sections 4 and 5 present the main assumptions of the exergy analysis and its results, while in Section 6, the conclusions are drawn. Further details on the model and the exergy analysis are provided in Appendix A, while additional results are presented in Supporting Information.

## 2 System Description

**Figure 1:**
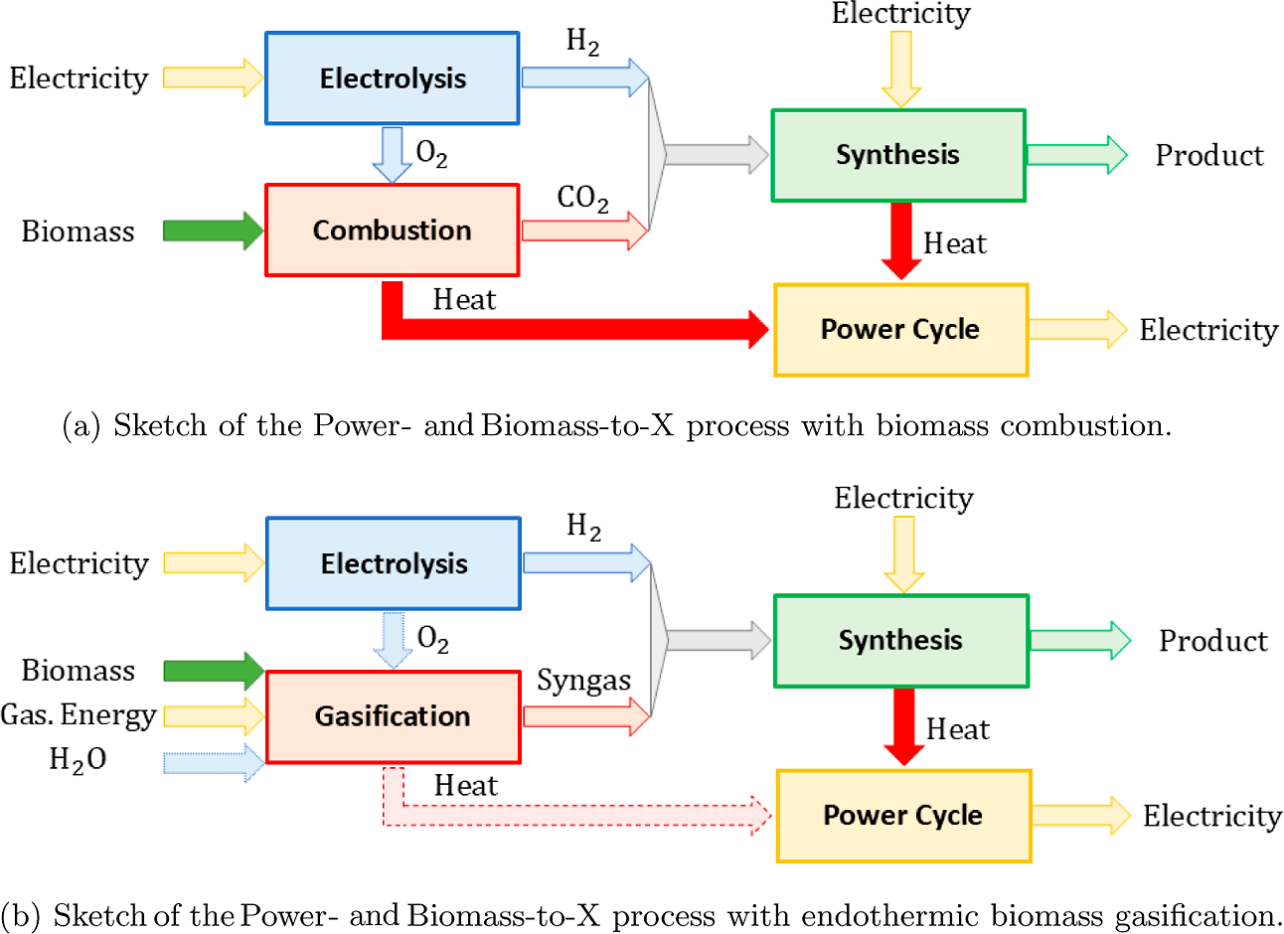
Block flow diagrams of Power- and Biomass-to-X processes using (a) combustion or (b) gasification (the considered gasification agents, i.e., water and oxygen, are marked with a dotted arrow). In case the gasification process is exothermic due to the operating conditions and the amount of supplied oxidizer, no additional energy input is needed and the produced heat can be supplied to the power cycle [dashed arrow in (b)]. Note: only the major material and energy streams are shown in the block-flowsheets.

The considered Power- and Biomass-to-X processes are composed of four key units, i.e., the biomass conversion, the water electrolysis, the synthesis unit, and the power cycle (see Figure 1).

Biomass is oxidized via either combustion or gasification using pure oxygen (the side-product of water electrolysis) or steam. The resulting stream is mostly composed of either CO_2_ and water (in case of combustion) or syngas, i.e., a mixture of mainly CO and H_2_ (in case of gasification). Although oxygen-based gasification generally occurs also in the presence of steam due to the residual moisture of biomass, the combination of oxygen and steam is not considered to isolate the effects of the pure oxidizing agents on syngas production and process efficiency. Also, the use of air as an oxidizing agent is not considered since nitrogen would dilute the CO_2_ or syngas stream, thus requiring an additional energy-intensive separation unit in real plants. Moreover, considering oxygen instead of air does not introduce any energy penalty, e.g., for air separation, since enough oxygen is produced as a side-product through water electrolysis.

For the combustion-based Power- and Biomass-to-X process, the purified CO_2_ stream at the outlet of the combustion unit is fed into the synthesis unit together with hydrogen produced by the low-temperature water electrolysis unit to adjust the H:C ratio of the mixture. The reactant stream is then converted to the product of interest in the synthesis unit, where it is separated from the side products. Similarly, for the gasification-based Power- and Biomass-to-X process, the purified syngas stream at the outlet of the gasification unit is supplied to the synthesis unit after adjusting the H:C ratio with additional hydrogen to synthesize the product of interest.

Additionally, the power cycle unit converts part of the high-temperature thermal energy of the exothermic units into electricity, thus reducing the net electricity demand of the overall Power- and Biomass-to-X process.

## 3 Model

The models of the Power- and Biomass-to-X process units, i.e., the biomass conversion, the water electrolysis, the synthesis, and the power cycle units, are based on steady-state mass and energy balances and are written in MATLAB. Ideal overall reaction conversion values (100%) and no side reactions are assumed within the units. This approach has the key advantage that the results are not affected by kinetics, whose performance depends on the chosen catalyst, reactor technology, and operating conditions, while it is still able to capture some inherent differences in efficiency between the considered processes. Furthermore, many real synthesis processes using selective catalysts approximate full conversion through recycling most of the unreacted reactants.^20^

The results obtained with these models represent the upper limits in performance determined by the thermodynamics of the processes. To verify and validate these results, a more detailed model has also been built in Aspen Plus for an example case study, i.e., Power- and Biomass-to-Methanol (see Section 5.3.4).

In the following subsections, the main modeling equations and assumptions are discussed. A summary of the key assumptions is reported in Table A2.

### 3.1 Biomass Conversion Unit

Biomass is converted to a suitable gaseous intermediate via either combustion or gasification to be fed into the synthesis unit. In the following, the models of the biomass feedstock and biomass conversion units are described.

#### 3.1.1 Biomass

For all the considered biomass conversion processes, the inlet biomass is assumed to be composed of C, H, O, N, and S atoms and represented by the pseudomolecule C_*x*_H_*y*_O_*z*_N_*w*_S_*t*_, where *x*, *y*, *z*, *w*, and *t* are the molar fractions calculated from the ultimate analysis. Woody biomass (ultimate analysis in Table 1) is considered the reference biogenic feedstock for the results in Section 5. Moreover, the feedstock is assumed to be dry and without ashes, and the energy demand for biomass pretreatment is neglected. These choices lead to the identification of an upper bound to the overall Power- and Biomass-to-X efficiency. In practice, the moisture in the biomass adds an energy penalty due to the heat required for its evaporation (heat from water condensation in the flue gas is generally not recovered), while flue gas cleaning including ash removal might also require an energy demand.

**Table 1:** Ash-Free Dry Biomass Composition in % wt (Ultimate Analysis)^21^

	*x*_C_	*x*_O_	*x*_H_	*x*_N_	*x*_S_
Woody biomass (mean)	52.1	41.2	6.2	0.4	0.1

The higher heating value (HHV_bio_) of the dry biomass is calculated via the correlation proposed by Sheng and Azevedo^22^
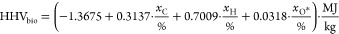
1where *x*_C_, *x*_H_, and *x*_O*_ are the mass fractions of carbon, hydrogen, and equivalent oxygen (sum of oxygen and other elements in the organic matter) from the ultimate analysis. The calculated HHV_bio_ of the dry ash-free woody biomass with the composition described above is 20.7 MJ/kg.

The lower heating value (LHV_bio_) is then obtained by subtracting the energy for water condensation at 25 °C. The estimated LHV_bio_ of such biomass is 19.3 MJ/kg.

#### 3.1.2 Combustion Unit

**Figure 2:**
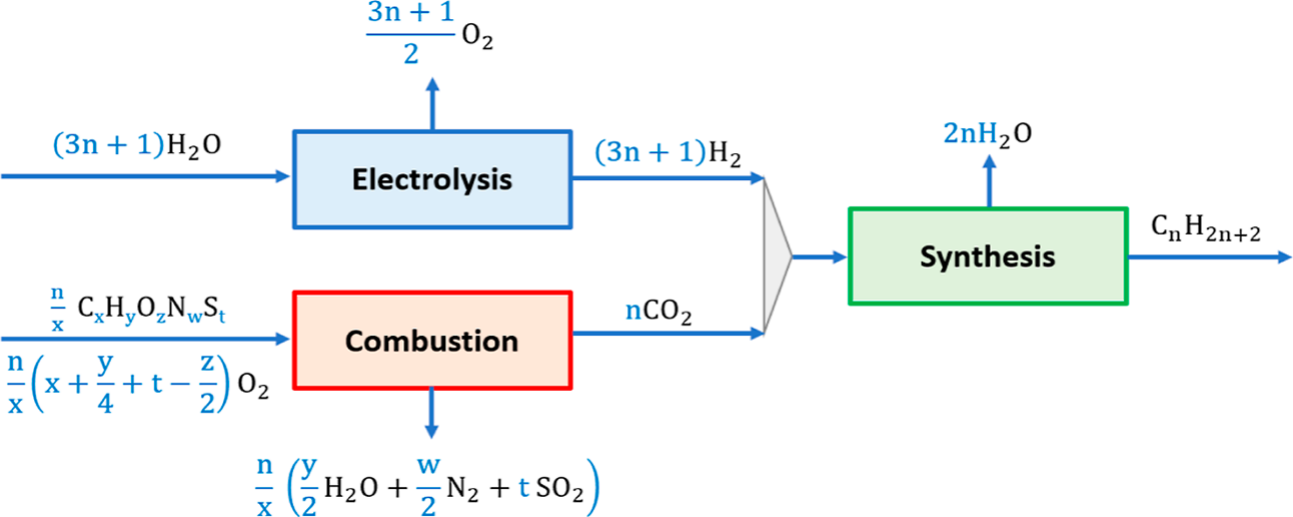
Molar flow rates in the Power- and Biomass-to-X process with biomass combustion for the production of alkanes. The stoichiometric coefficients are shown in blue.

In the model of the combustion unit, biomass is completely oxidized. The carbon, hydrogen, and sulfur atoms are oxidized to CO_2_, H_2_O, and SO_2_, respectively, while the nitrogen atoms form N_2_ (see Table A1 for the reaction and Figure 2 for a sketch summarizing the molar flows).

The oxygen contained in the biomass is used for the oxidation of biomass. Any additional oxygen that is needed to achieve the desired conversion is provided by the electrolysis unit (Subsection 3.2). In fact, the model shows that the electrolysis unit can always supply enough oxygen for the considered processes, although the electrolysis unit aims to satisfy the hydrogen demand.

The maximum temperature assumed in the combustion unit is 1000 °C. The relatively low combustion temperature is motivated by the generally low melting point of biomass ashes. As the flame temperature of stoichiometric oxy-combustion is too high, a recycle of cold product gases within the unit is assumed to reach the desired combustion temperature. Nevertheless, the choice of the combustion temperature value does not affect the energy efficiency of the modeled unit but only the exergy flows. In fact, the energy efficiency of the combustion unit defined as the ratio between the energy output, i.e., the generated heat, and the energy input, where the chemical energy of biomass is estimated via the LHV, is equal to 100% (see Figures S5–S8 in Supporting Information) due to the considered modeling assumptions, e.g., ideal components and reactions, no compression energy for cold product gas recycle, and no heat losses. In contrast, when nonidealities are considered as, e.g., in the detailed Power- and Biomass-to-Methanol model (see Section A.6), lower energy efficiencies are achieved.

The produced CO_2_ is then separated from N_2_, SO_2_, and H_2_O before being supplied to the synthesis unit. No energy demand for CO_2_ separation is considered in the energy balance. In real processes, SO_2_ can be mostly removed with H_2_O via condensation, while the small amount of N_2_ can be either separated or not since it is generally an inert gas in CO_2_ conversion processes. Furthermore, the need for separation might also depend on the tolerance of the catalyst of the downstream synthesis unit.

The whole energy content of biomass is transferred into the power cycle (see Figure 1a and Subsection 3.4) by cooling the flue gases from the operating to the ambient temperature. The CO_2_ stream, which is assumed to be at ambient conditions at the interface with the synthesis unit, has no useful energy content (LHV = 0). Further details and assumptions on the energy balance can be found in Appendix A.2.

#### 3.1.3 Gasification Unit

**Figure 3:**
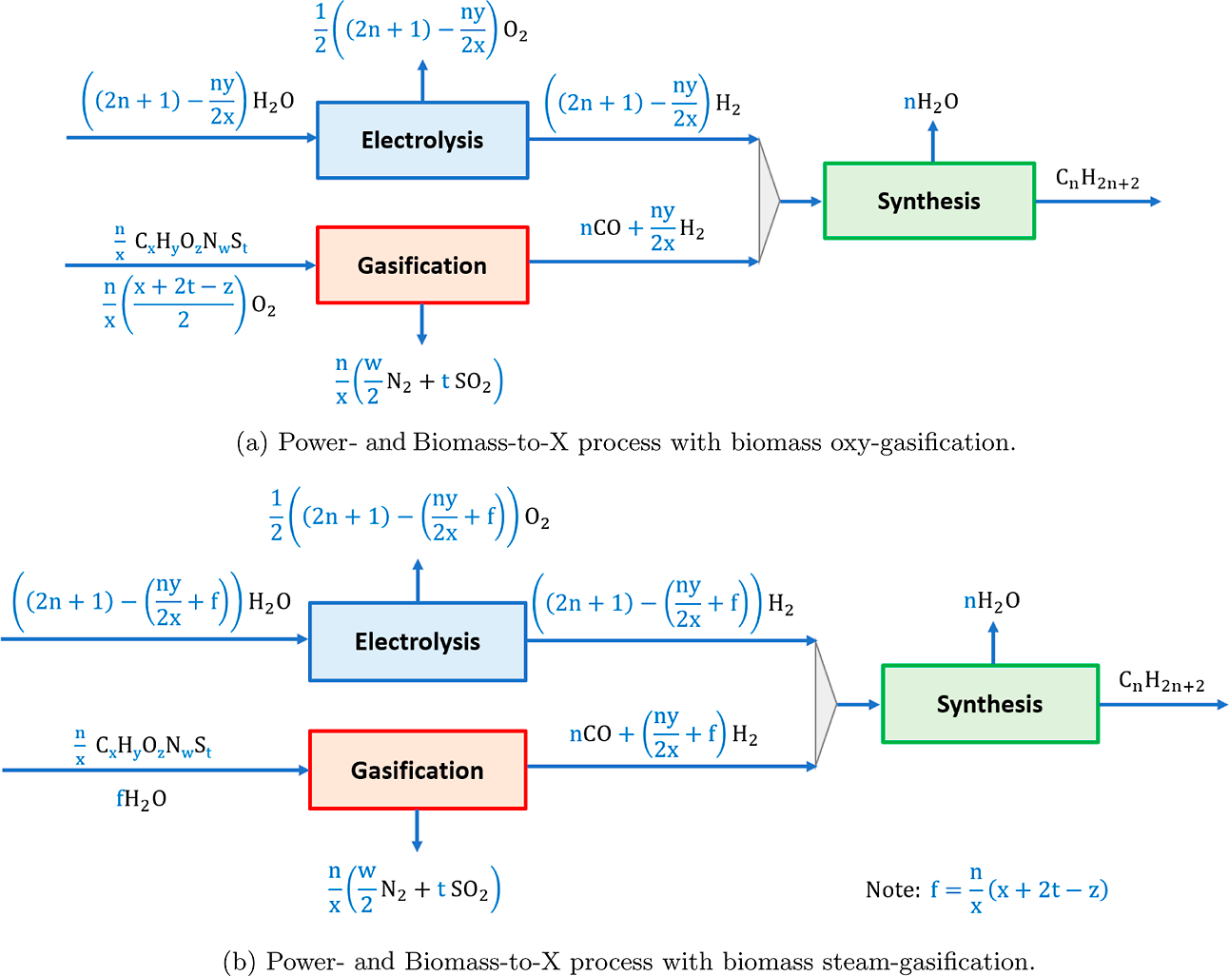
Molar flow rates in the Power- and Biomass-to-X process with biomass oxy-gasification (a) and steam-gasification (b) for the production of alkanes. The stoichiometric coefficients are shown in blue.

In the model of both oxygen-based and steam-based gasification units, we assume that all the carbon and hydrogen atoms in the biomass are converted to CO and H_2_, while nitrogen and sulfur atoms form N_2_ and SO_2_. However, how carbon is oxidized differs among the two considered gasification processes. In particular, in the model of oxygen-based gasification, oxygen is added to partially oxidize all the carbon atoms (Reaction 2). In the model of steam-based gasification, Reaction 2 occurs until the oxygen content in the biomass is depleted. Steam is then added to partially oxidize the remaining carbon and generate hydrogen, as in Reaction 3. The overall conversion reactions and a sketch summarizing the molar flows for the two case studies can be found in Table A1 and Figure 3, respectively.
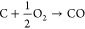
2
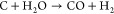
3

Depending on the biomass composition, operating conditions, and assumed conversion, biomass gasification can be exothermic or endothermic. If the biomass gasification process is endothermic (the typical operating condition), an energy input  has to be supplied to drive the process as shown in the energy balance below:
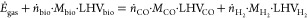
4where ṅ and *M* are the molar flow rates and the molecular weights, respectively. Instead, if the gasification process is exothermic, biomass represents the only energy input, and the unit produces high-temperature heat that is supplied to the power cycle (see Figure 1b and Subsection 3.4).

Among the two considered gasification processes, steam-based gasification is expected to have a higher energy demand (when endothermic) than oxygen-based gasification because Reaction 3 is endothermic. The whole energy demand , including heat for steam production, is considered to be supplied via electricity as the assumed gasification temperature is relatively high (900 °C, a typical value for biomass gasification^14^). Alternatively, other high-temperature heat sources, e.g., heat from the combustion of purge streams or solar energy stored in molten salts, could have been used. In contrast, the more efficient reversed Brayton–Joule cycles or heat pumps cannot commercially achieve the target temperature of 900 °C.^23^ Heat for steam production could also be supplied via heat integration with the synthesis unit, but this option has not been considered.

Similarly to the combustion unit, no energy demand for separating N_2_ and SO_2_ from H_2_ and CO is considered. More details about the models and energy balances can be found in Appendix A.2.

### 3.2 Electrolysis Unit

The electricity demand (*P*_el_) is calculated by assuming a first law efficiency of the electrolyzer equal to η_el_ = 0.6, a typical value for the mature low-temperature electrolysis technology,^24^ as follows:
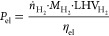
5The electrolysis unit is operated at 60 °C. It produces excess heat since it is operated above the thermoneutral voltage as in conventional operating conditions. However, we assume that this excess heat is wasted and is not used for electricity production due to its low temperature level. In reality, it could be used for biomass drying, for district heating, or upgraded with respect to temperature for industry applications.^25^

The nominal hydrogen flow rate, which also affects the size of the electrolysis unit, depends on the considered pathway (gasification or combustion), target product, and biomass composition since it is calculated according to the stoichiometry of the synthesis reactions. In particular, the gasification pathway requires a lower hydrogen flow rate (compare Figures 2 and 3).

### 3.3 Synthesis Unit

The inlet stream of the synthesis unit is a CO_2_–H_2_ mixture or syngas for the biomass combustion and gasification pathway, respectively. The product is then synthesized according to the reaction stoichiometry by assuming ideal conversion and no side reactions. For many products, real processes have been proposed that achieve very high selectivity and almost full conversion either in a single pass or by recycling unreacted reactants.

We consider the synthesis of four target products with significantly different C:H:O ratios and with high relevance for the chemical and transport sectors: the simplest alkane (methane), alcohol (methanol), and ether (dimethyl ether) and a long-chain hydrocarbon representing the kerosene range of hydrocarbons (dodecane).

The synthesis reaction changes according to the feed stream composition (CO_2_–H_2_ mixture or syngas) and the considered product. For alkanes, the overall synthesis reactions from a CO_2_–H_2_ mixture and syngas are
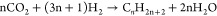
6
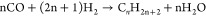
7The synthesis reactions for the other products can be found in Table A1.

The exothermic synthesis reactions are assumed to occur at a typical (constant) reaction temperature. In particular, the considered reaction temperature is 400 °C for methane,^26^ 250 °C for methanol,^26^ 300 °C for dimethyl ether,^27^ and 250 °C for dodecane^9^ synthesis via the low-temperature Fischer–Tropsch process.

The energy demand for compression of the reactants up to a typical reaction pressure is considered. In particular, the considered reaction pressure is 10 bar for methane,^26^ 75 bar for methanol,^10,26^ 10 bar for dimethyl ether,^27^ and 25 bar for dodecane.^9^ The electric energy demand is estimated by simulating a multistage compressor with intercooling in Aspen Plus. The heat removed in the intercooling section of the multistage compressor is not recovered since there is no net heat demand within the synthesis unit and the average temperature level of the heat is relatively low (ca. 100 °C).

In contrast, no energy for product separation, e.g., via distillation, is considered in the energy balance. This assumption allows for identifying a thermodynamic efficiency limit not dependent on a specific separation process. Moreover, neglecting this contribution does not lead to a large overestimation of process efficiencies since the exergy efficiency of product synthesis is generally high (ca. 80–90% as calculated by Bongartz et al.^20^ with more detailed models of the production process of methane, methanol, and dimethyl ether from CO_2_–H_2_). Further information and assumptions about the energy balances can be found in Appendix A.2.

### 3.4 Power Cycle Unit

For all of the considered pathways, the heat from the main exothermic units is converted into electricity in a power cycle unit, thus reducing the net electricity demand of the electrolysis unit. The first-law efficiency of 40% and 30% are considered for the high-temperature (combustion and gasification unit) and medium-temperature (synthesis unit) heat source, respectively. These efficiencies are comparable to the efficiency of a coal power plant and a steam cycle using a high-quality residual heat source,^28^ respectively.

The thermal energy from water electrolysis and water condensation in the products is instead not recovered and converted into electricity due to its low exergy content.

## 4 Exergy Analysis

An exergy analysis of the Power- and Biomass-to-X processes is carried out to compare the biomass combustion and gasification pathways and identify the units that are mainly responsible for energy degradation. The exergy balance of each process unit can be written as follows:
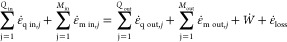
8where  and  are the exergy flows related to heat flows and material streams, respectively, Ẇ is the work exchange, and  the exergy losses for the considered unit. In this work, the term  accounts for not only the irreversibilities, e.g., mixing of the reactants, but also for what is not considered “useful effect”, i.e., exergy in the side products.

**Figure 4:**
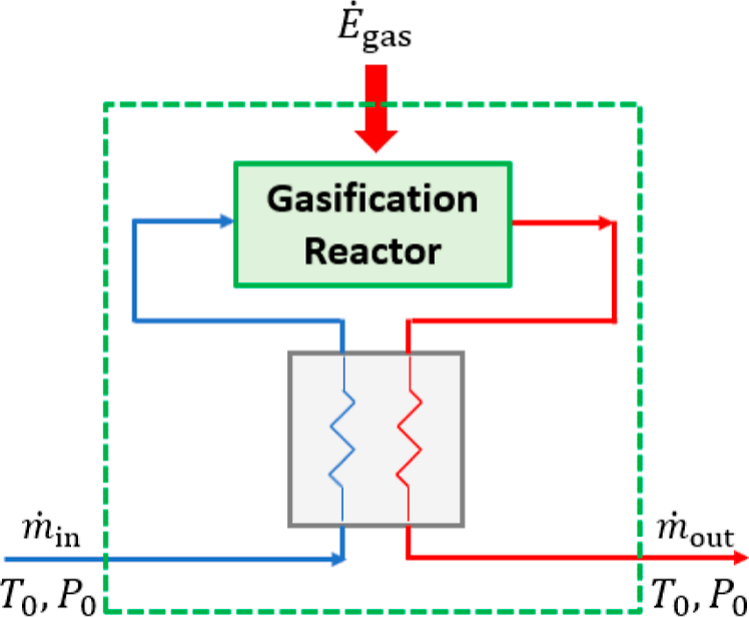
Heat integration for the (endothermic) gasification unit, contained in the dashed green line, is shown. Similar heat integration was considered for the electrolysis and synthesis units.

The exergy flow related to heat flows  is calculated by considering the typical reaction temperature of each unit as described in Appendix A.4.1. In contrast, the exergy flows due to the preheating of the reactant stream and the cooling of the product streams are not considered since perfect heat integration is assumed to minimize the energy demand and the exergy loss (see Figure 4 and Appendix A.2 for more details on heat integration of the units).

The exergy flow related to material streams  is composed of four contributions, i.e., kinetic, potential, physical, and chemical. The kinetic and potential contributions are often negligible, and the physical contribution, related to the temperature and pressure of the stream, is null since all the streams at the interface of the unit are for simplicity assumed to be at ambient conditions (*T*_0_ = 298 K, *P*_0_ = 1 bar). Thus, the most relevant contribution of  is the chemical one, which is calculated as in Appendix A.4.2.

The chemical exergy related to the oxygen and water feed streams is also considered. The recycle of the produced water in the synthesis and combustion units and of oxygen reduces the net exergy demand of reactants , although it plays a marginal role in the overall process efficiency. In contrast, the exergy demand for separating the target product from the side products is neglected.

The overall exergy efficiency for the Power- and Biomass-to-X processes is calculated as follows:
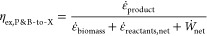
9where  is the exergy content of the product,  is the exergy content of biomass,  is the exergy content of the other reactants (water and oxygen), and  is the net electricity demand, which also includes heat for gasification  in case it is endothermic.

## 5 Results and Discussion

In the following, the results of the exergy analysis of the considered Power- and Biomass-to-X processes are presented. Furthermore, a sensitivity analysis of the key parameters is performed, and the key aspects of the practical implementation of these processes are discussed. The results of a similar analysis based on energy rather than exergy are shown in the Supporting Information for the sake of completeness.

### 5.1 Base Case

The exergy efficiency of the Power- and Biomass-to-X processes is calculated for the considered products by assuming woody biomass as carbon feedstock. For the considered models, which combine nearly ideal assumptions, e.g., no side reactions and perfect heat integration, and empirical efficiency parameters (see Section 3 and Table A2), the calculated exergy efficiencies represent an efficiency benchmark for Power- and Biomass-to-X processes modeled in detail.

**Figure 5:**
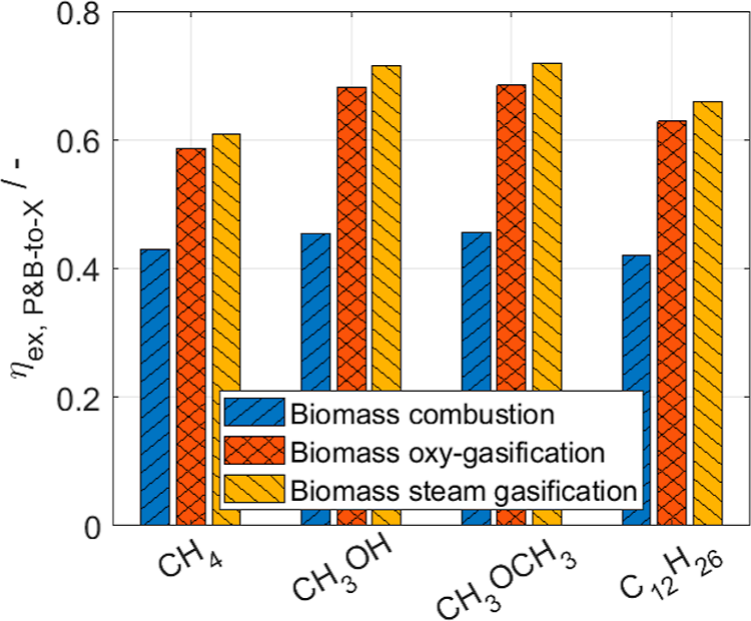
Exergy efficiency of Power- and Biomass-to-X processes with biomass combustion and gasification for different products X. Woody biomass is considered as carbon feedstock.^21^

Among the different target products, the production processes of methane and dodecane have the lowest exergy efficiency (Figure 5). The main reason is that their production needs the highest number of hydrogen moles per mole of carbon (see Table A1). In contrast, the production processes of methanol and dimethyl ether have the highest exergy efficiency since a lower number of hydrogen moles is needed based on the reaction stoichiometry (see Table A1).

**Figure 6:**
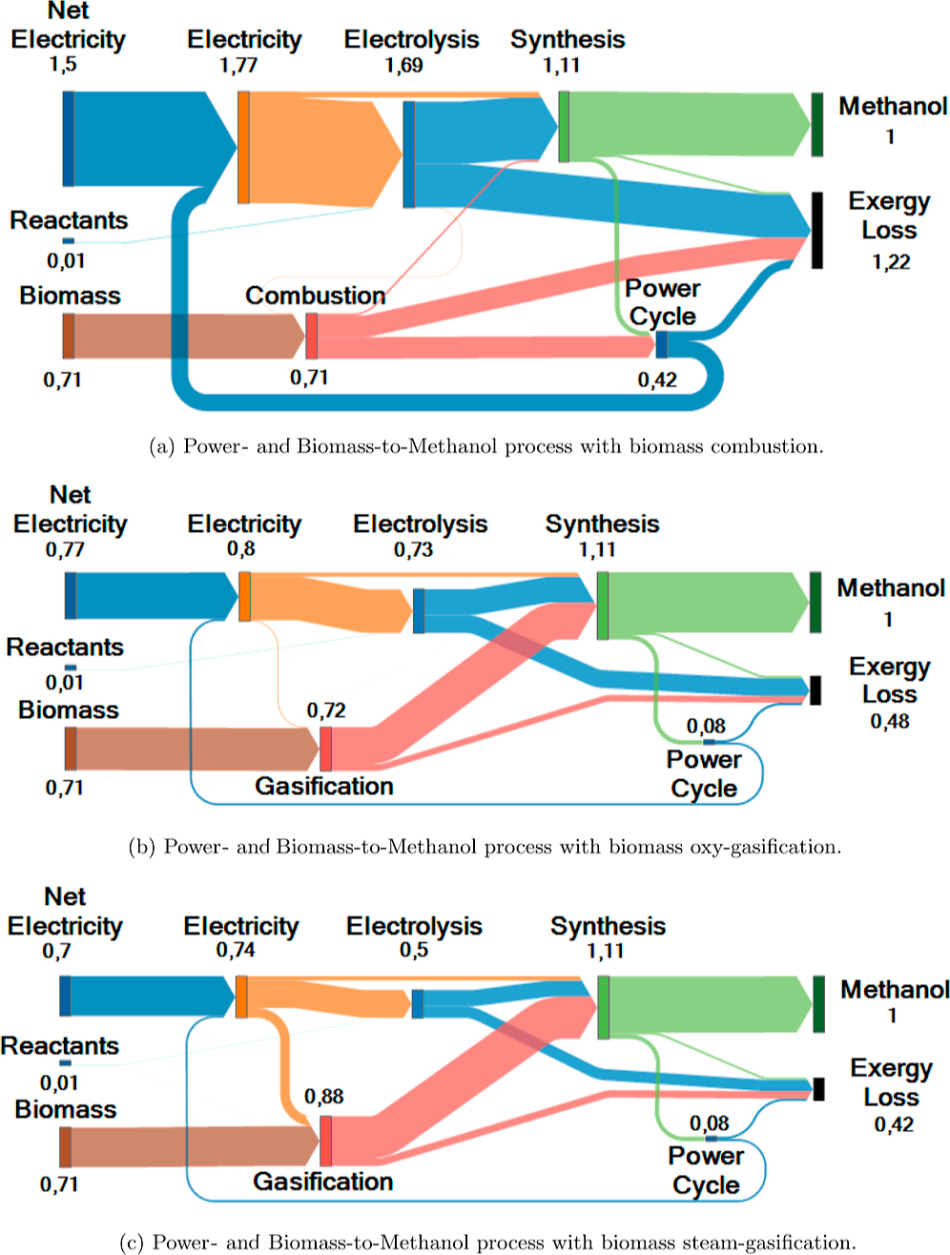
Sankey diagrams of the exergy flows for a Power- and Biomass-to-Methanol process with (a) biomass combustion, (b) biomass oxy-gasification, and (c) biomass steam-gasification. The exergy flows in MW are scaled per unit of product. The numbers at the diagram nodes represent the exergy flows of the energy sources and the total exergy flows through the process units.

The results also show that gasification-based Power- and Biomass-to-X processes are significantly more efficient than combustion-based ones (around 15–20 percentage points) under the considered assumptions (Figure 5). A part of this efficiency difference is due to the high exergetic losses of the combustion unit: although the energy efficiency is assumed to be 100% (no heat losses; see Figures S5–S8 in Supporting Information), the conversion of chemical to thermal energy causes significant energy degradation, resulting in an exergy efficiency of the combustor lower than 55% (see Figure 6a for Power- and Biomass-to-Methanol). In contrast, gasification processes avoid the conversion of high-quality chemical energy into lower-quality thermal energy since the energy of biomass is transferred into the produced syngas, thus leading to higher exergy efficiencies (around 85% in Figure 6b and 6c). Furthermore, gasification-based processes can already produce part of the needed hydrogen for the downstream synthesis unit and do not require additional hydrogen to convert CO_2_ into CO due to the different reaction stoichiometry. The combination of these two effects significantly reduces the amount of hydrogen supplied by the electrolyzer, which is responsible for large exergy loss, and thus the energy input that is supplied via electricity. In fact, the biomass combustion pathway has a significantly higher (>45% for methanol production) net electricity input. The higher electricity production in the power cycle of the combustion-based pathway cannot offset this higher electricity demand of the electrolyzer, thus leading to a lower overall exergy efficiency than gasification pathways.

Among the considered gasification processes, steam-based gasification processes seem to be more efficient since syngas with a higher H_2_:CO ratio can be produced despite the higher energy demand to drive the gasification process. The efficiency difference is mainly due to the lower exergy losses in the electrolysis unit (ca. 30% lower hydrogen demand for Power- and Biomass-to-Methanol). In contrast, the exergy efficiency of the gasification unit is similar for the two case studies (around 85%). The potential of steam-based gasification might even still be underestimated by the model since part of the heat needed for steam production is supplied via heat integration by the exothermic synthesis unit. The result that steam-based gasification is more efficient than oxygen-based gasification aligns with the findings of Brown et al.^15^ who compared gasification processes with different gasifying agents for electricity production: the exergy efficiency of the gasification unit using oxygen and steam as gasifying agents does not significantly differ (ca. 73–77%). The lower efficiencies are due to the more detailed models and different operating conditions in Brown et al.^15^ For the sake of completeness, the molar, energy, and exergy flows are shown for all products in the Supporting Information.

### 5.2 Sensitivity Analyses

The effect of key process parameters on the efficiency of Power- and Biomass-to-X processes with biomass combustion and gasification is investigated. In particular, the impact of the efficiency of the electrolysis unit, the efficiency of the power cycle unit, and the composition of the carbon feedstock on the exergy efficiency is analyzed.

#### 5.2.1 Sensitivity Analysis: Electrolyzer Efficiency

The efficiency of the electrolysis unit affects significantly the efficiency of the overall Power- and Biomass-to-X process since this unit is responsible for high exergy losses. Increasing the electrolyzer efficiency from 60 to 80% increases the Power- and Biomass-to-X efficiency of all products by around 10 percentage points (see Table A5). As expected, the ranking of the pathways in terms of the exergy efficiency does not change with increasing electrolyzer efficiency. However, the gap between the combustion and the gasification pathways is narrower when the efficiency of the electrolyzer is higher since combustion-based processes have higher hydrogen demands. Among the different products, methane production benefits the most from the electrolyzer efficiency improvement since it requires the highest number of hydrogen moles per mole of carbon.

Interestingly, the difference in efficiency between the steam-based and oxygen-based gasification processes becomes smaller with higher electrolyzer efficiencies (Table A5). This is due to the lower exergy losses in the electrolysis unit for hydrogen production in both absolute and relative terms. If the electrochemical hydrogen production route is sufficiently efficient, the use of steam gasification as a way to produce additional hydrogen at the price of a higher energy demand for gasification might not be worth it. However, this situation does not happen with the current state-of-the-art low-temperature water electrolyzers. Furthermore, in the case steam was produced by recovering heat from the synthesis unit (not considered here), even higher electrolyzer efficiencies would be needed to make oxygen-based gasification processes more efficient than steam-based ones.

#### 5.2.2 Sensitivity Analysis: Power Cycle Efficiency

The efficiency of the power cycle unit plays a minor role compared to the efficiency of the electrolyzer: a better conversion of the residual heat into electricity leads to little improvements in the Power- and Biomass-to-X exergy efficiency (see Table A6) since the power cycle unit handles a limited amount of energy, and most of the exergy loss has already occurred in the upstream units as shown in Figure 6.

Among the different pathways, the efficiency of gasification-based Power- and Biomass-X processes is slightly less dependent on the efficiency of the power cycle unit since less heat is provided to that unit.

#### 5.2.3 Sensitivity Analysis: Carbon Source Composition

**Figure 7:**
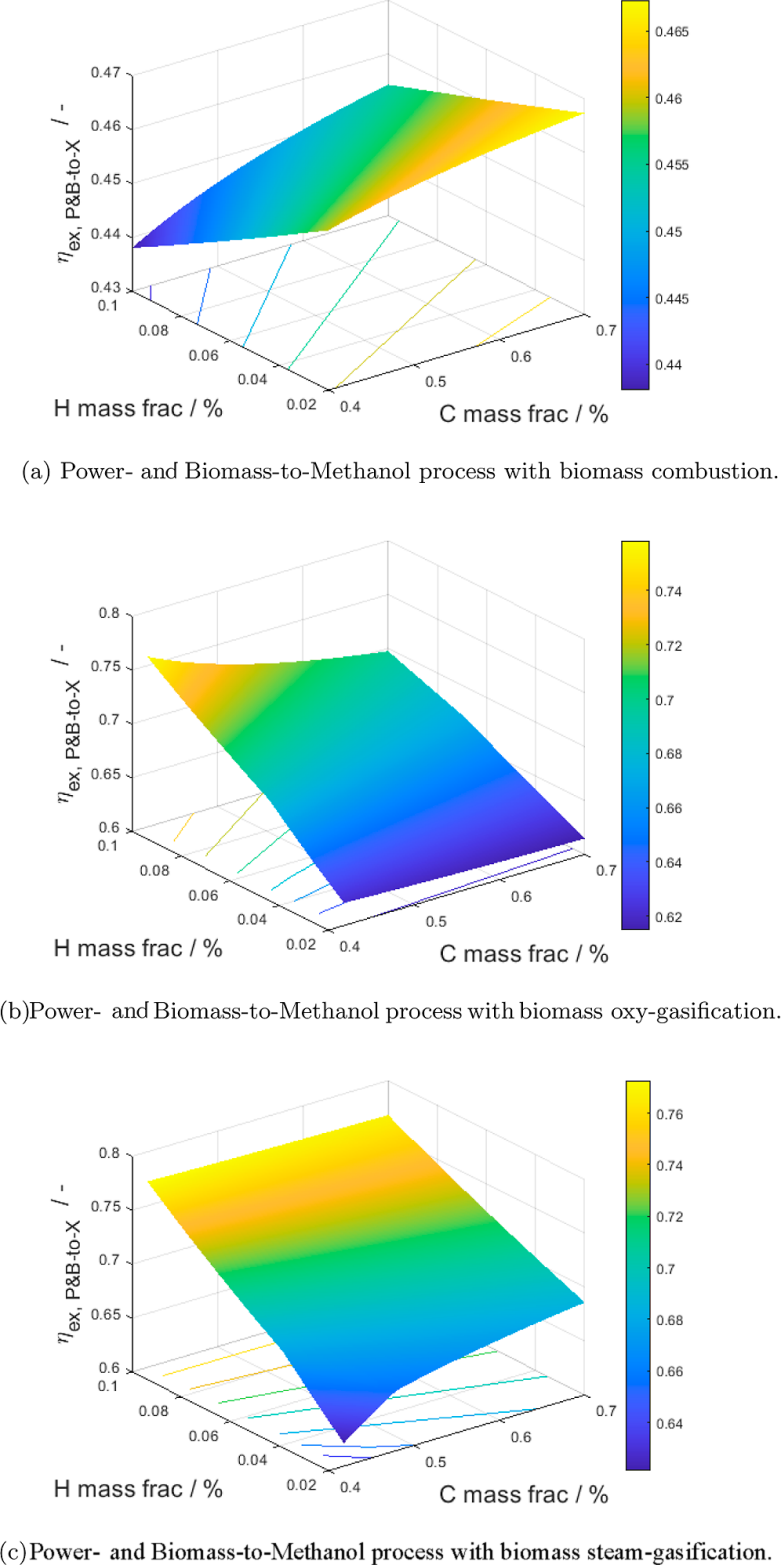
Sensitivity analysis of the exergy efficiency of a Power- and Biomass-to-Methanol process with (a) biomass combustion, (b) biomass oxy-gasification, and (c) biomass steam-gasification, with respect to the feedstock composition. The mass fractions of S and N were kept constant to 0.001 and 0.004, respectively (values for woody biomass); the mass fractions of C and H were varied, while the oxygen content was calculated by difference. Note: due to the overstoichiometric presence of oxygen in the biomass at low carbon contents (lower than ca. 0.42), no efficiency could be calculated for the gasification-based processes with the considered model.

The composition of the carbon feedstock has an important impact on the exergy efficiency of Power- and Biomass-to-X processes since it affects how much heat is produced or required in the combustion and gasification unit and how much hydrogen can be potentially produced via gasification. In Figure 7, we show the exergy efficiency of combustion-based and gasification-based Power- and Biomass-to-Methanol processes, respectively, by varying the carbon and hydrogen mass fractions in the biomass. Relatively wide ranges ([0.4, 0.7] and [0.02, 0.10] for carbon and hydrogen, respectively) are considered since the sustainable feedstock composition, e.g., biomass, biogas, and wastes, can vary significantly.^21,29^

The sensitivity analysis shows that gasification-based processes are more efficient, irrespective of the biomass composition. The changes in the exergy efficiency for the combustion-based processes are smaller than the ones for the gasification-based processes when considering the same H and C ranges. Therefore, the choice of a proper biomass feedstock is more important for gasification processes. Furthermore, according to the model results, the composition of the most suitable feedstock differs for the three considered processes.

For combustion-based processes, high carbon contents in the feedstock increase the amount of product per mole of biomass as well as the LHV of biomass. These two aspects conflict with each other since they contribute to the numerator and denominator of the exergy efficiency, respectively (see Section 4); nevertheless, the exergy efficiency increases with high carbon contents in the feedstock (Figure 7a) since the increase of produced product plays the major role. In contrast, high hydrogen contents in the feedstock seem not to be worth it since hydrogen only contributes to the LHV of biomass, whose energy content is mostly wasted and not entirely converted into electricity in the power cycle unit. In fact, the reduction of the net electricity demand due to the additional electricity produced in the power cycle is smaller than the increase in the energy stored in the biomass per unit of product when increasing the biomass LHV via hydrogen concentration.

**Figure A1:**
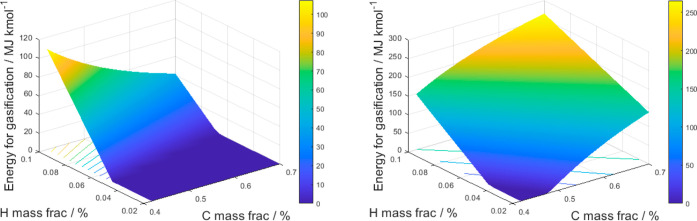
Sensitivity analysis of the heat demand for gasification of the Power- and Biomass-to-Methanol process with respect to the feedstock composition using oxygen (left) and steam (right) as gasifying agents. The mass fractions of S and N were kept constant at 0.001 and 0.004, respectively (values for woody biomass); the mass fractions of C and H were varied, while the oxygen content was calculated by difference. When the energy demand for gasification is equal to 0, the gasification process is exothermic.

For oxygen-based gasification processes, high hydrogen mass fractions increase the exergy efficiency since, according to the model, they increase the amount of H_2_ produced per mole of carbon within the gasification unit, thus reducing the hydrogen demand from the electrolysis unit (Figure 7b). In contrast, high carbon concentrations slightly decrease the overall Power- and Biomass-to-Methanol efficiency since the exothermic reaction of carbon oxidation (Reaction 2) is promoted and the H:C ratio is reduced. Higher exergy efficiencies are achieved when the biomass composition is such that the gasification process is endothermic (see Figure A1) since endothermic processes have lower exergy losses.

**Figure 8:**
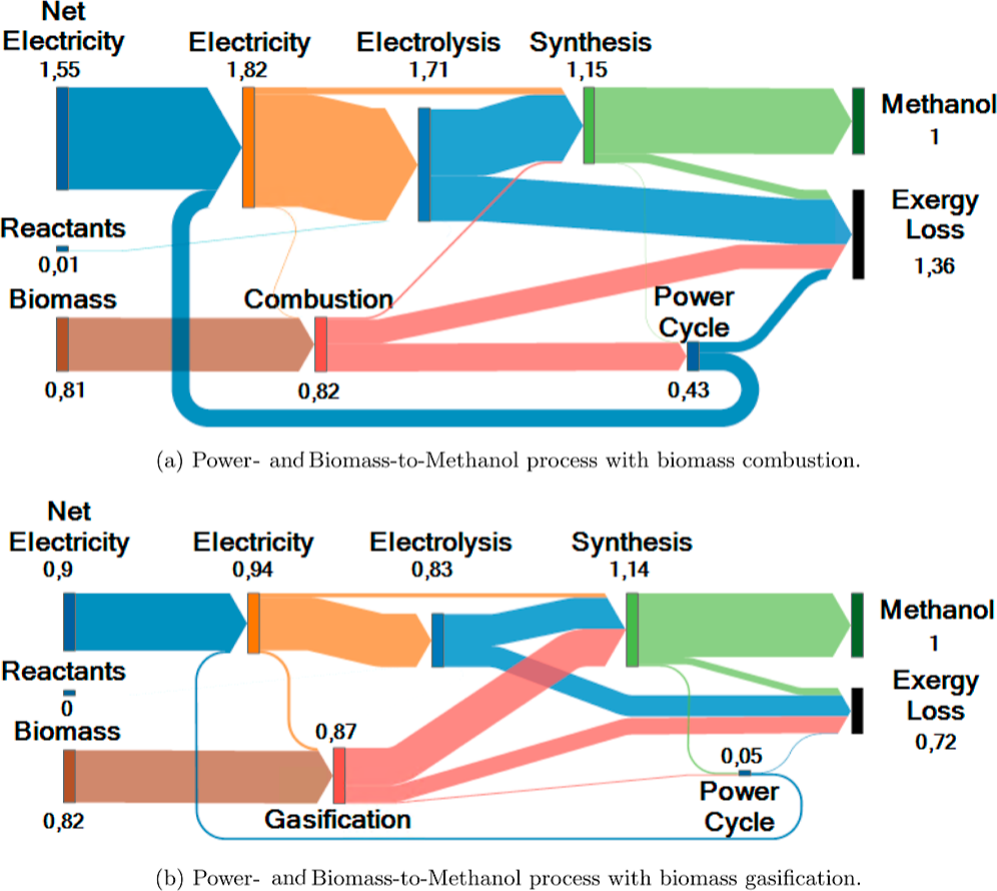
Sankey diagrams of the exergy flows for a Power- and Biomass-to-Methanol process with (a) biomass combustion and (b) biomass gasification modeled in detail in Aspen Plus. The exergy flows in MW are scaled per unit of product. The numbers at the diagram nodes represent the exergy flows of the energy sources and the total exergy flows through the process units. Note: these Sankey diagrams differ from the ones in Figure 6 for the level of detail of the models (complex vs simple models, respectively).

**Figure 9:**
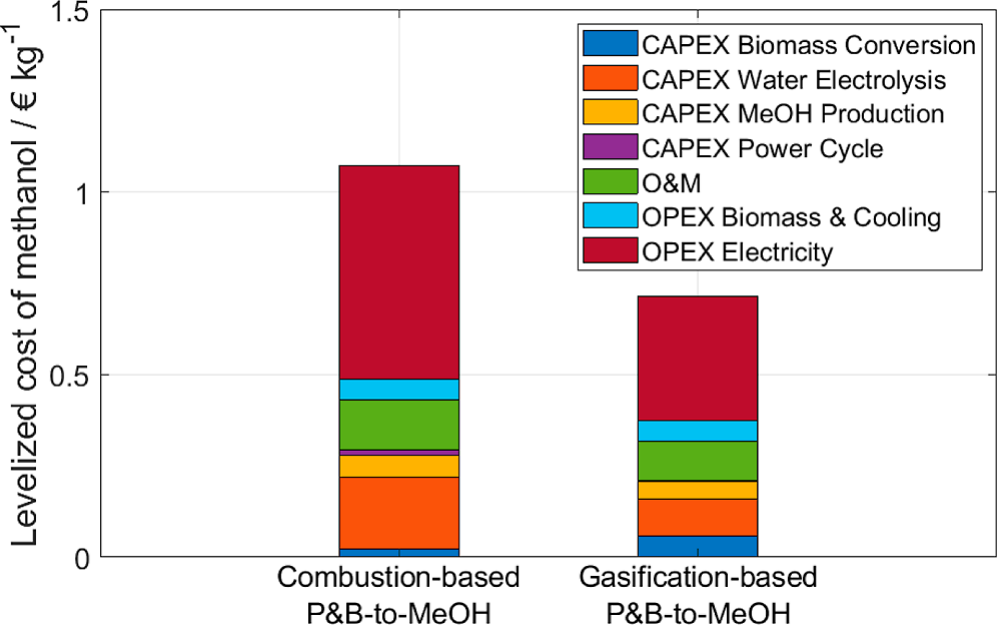
Levelized cost of methanol produced via the combustion-based and gasification-based pathways. Note that a mixture of oxygen and steam is considered as the gasifying agent as discussed in Section A.6.

For steam-based gasification processes, an increasing concentration of hydrogen and carbon improves the exergy efficiency of the Power- and Biomass-to-Methanol process (Figure 7c) since it leads to higher hydrogen production in the syngas. In particular, high carbon concentrations reduce the oxygen content in the feedstock and require higher amounts of steam, thus promoting the endothermic reaction of carbon oxidation via steam (Reaction 3) according to the considered model. Similarly to the oxygen-based gasification process, higher exergy efficiencies are achieved when the gasification process is endothermic (Figure A1).

Finally, although sensitivity analyses on feedstock composition provide interesting insights into the variation of the exergy efficiency of Power- and Biomass-to-X processes, the feedstock composition cannot be chosen arbitrarily. For this reason, the efficiency of Power- and Biomass-to-X processes has also been calculated for real carbon feedstock compositions. Similarly to the observations above, it was shown that different carbon feedstocks are more suitable for gasification-based rather than combustion-based processes (the results can be found in Section 2 of the Supporting Information). Furthermore, it is important to highlight that other practical aspects of biomass such as its moisture and sulfur and ash content (not considered in this analysis) can significantly affect its suitability for efficient use in Power- and Biomass-to-X processes since an energy-intense biomass pretreatment and flue gas post-treatment might be needed.

### 5.3 Practical Considerations

The calculated exergy efficiencies represent an upper thermodynamic limit for the considered Power- and Biomass-to-X processes (except for the electrolyzer and power cycle, where current practical values were used). In fact, the efficiency of real processes also depends on other components in the carbon feedstock such as ashes and moisture, the energy demand of auxiliaries and separation units, nonidealities in reactors (e.g., side product formation), and imperfect heat integration. Therefore, the efficiency of real processes is lower, and the efficiency gap between the combustion-based and gasification-based Power- and Biomass-to-X processes might change.

In the following, a critical analysis of how such practical considerations might impact the efficiency of Power- and Biomass-to-X processes is carried out. Furthermore, a comparison between a combustion-based and gasification-based Power- and Biomass-to-X process modeled by accounting for most of the process nonidealities is presented.

#### 5.3.1 Challenges of Biomass as Feedstock

As discussed above, biomass is a promising carbon feedstock for the synthesis of valuable and sustainable products since, differently from CO_2_, it also carries energy into the production process. Furthermore, biomass utilization for product synthesis is particularly effective when hydrogen from electrolysis is added. In fact, adding electrolytic hydrogen to Biomass-to-X processes increases the carbon yield and the process efficiency (although the latter depends on the electrolyzer efficiency), as already shown for instance for Power- and Biomass-to-Liquid processes.^10,11^

However, biomass handling requires energy-intense pretreatment, e.g., biomass drying and particle size reduction,^30,31^ to increase the conversion performance and the quality of the produced syngas.^14,31^ Furthermore, elements like sulfur, chlorine, and potassium can contribute to plant corrosion and slag formation when biomass is converted.^32^ Moreover, ashes and the formed pollutants, e.g., SO_*x*_ and NO_*x*_, have to be properly removed since they cause fouling and might poison the catalysts of the downstream synthesis unit.^12^

Finally, biomass is not uniformly distributed, and its availability might be limited in certain areas. However, biomass transport over long distances is generally not economically viable due to its relatively low energy density. Therefore, the size of the conversion plants is generally relatively small, and the plant location and biomass supply chain should be already considered in the design phase to minimize the costs and the global warming potential.^33^

#### 5.3.2 Combustion Processes

Our model of combustion processes does not differ significantly from real processes since biomass oxidation is generally almost complete if conducted at proper conditions, e.g., good biomass-oxidizer mixing, combustion in stages, and high residence times.^32^ While excess oxygen could be used to ensure almost complete char combustion and CO burnout, its presence in the flue gases might hinder the direct feed of the clean and dried CO_2_ stream into the synthesis unit, since oxygen is poisonous for most of the catalysts. In that case, an energy-intensive separation process, such as a carbon capture unit, that separates CO_2_ from the flue gas should be considered. Furthermore, big recycles of the flue gases mainly composed of CO_2_ and H_2_O are needed to control the combustion temperature due to the high adiabatic flame temperature of oxy-combustion.

Combustion-based Power- and Biomass-to-X processes might require additional conversion units according to the considered target product and the catalyst for its synthesis. In fact, several catalysts are developed for CO since it is more active than CO_2_, thus meaning that an intermediate reverse water gas shift unit might be needed to reduce CO_2_ to CO before feeding the stream into the synthesis unit. However, catalysts allowing direct hydrogenation of CO_2_ have also been developed, e.g., for methanol production.^34^

#### 5.3.3 Gasification Processes

Real gasification processes deviate from the considered model in that the carbon conversion of biomass is not complete (conversion lower than 98% and dependent on the technology and the operating conditions^14^) due to the scarcity of the oxidizing agent. Furthermore, the selectivity to syngas is lower than what is predicted by the stoichiometry and varies according to the feedstock composition and particle size and the operating conditions such as temperature, pressure, and oxidant-to-biomass ratio.^7,35^ In fact, relevant amounts of side products, e.g., H_2_O, CO_2_, CH_4_, and tars are formed, and energy-intense and expensive flue gas cleaning processes might be required to remove impurities.^14,15,36^ However, their formation can be reduced by using catalysts, e.g., dolomite and alkali catalysts,^7,12,37^ and proper operating conditions.^14,37^

Although herein we investigate the gasification processes with pure oxygen and steam using simple models, these models are still able to capture key trends. In fact, steam-based gasification can effectively produce syngas with higher hydrogen content and energy density^12−14^ at the expense of a higher energy input. Nevertheless, the energy demand for gasification can be higher than what is predicted by the model due to high biomass moisture, excess steam that is generally injected, and nonideal heat integration.

Furthermore, the amount of CO_2_ in the syngas could be quite significant, especially if the oxidizing agent is fed in excess to reduce unconverted carbon and the high-temperature heat demand for gasification. The CO_2_ can be inert in the product synthesis process, depending on the catalyst. If required, the CO_2_ concentration could be reduced via separation processes, e.g., membranes and adsorption processes, and the separated CO_2_ could be recycled into the gasifier to increase the carbon efficiency and reduce emissions.

All the mentioned aspects reduce the efficiency of gasification-based Power- and Biomass-to-X processes and thus the efficiency gap with combustion-based processes.

#### 5.3.4 Validation with Detailed Models for Power- and Biomass-to-Methanol

As discussed in the previous sections, real processes pose additional challenges such as incomplete reactant conversion, which were not considered in the process models described in Section 3. In this subsection, we show results for a combustion-based and gasification-based Power- and Biomass-to-Methanol process whose model accounts for several real aspects such as the presence of moisture in the biomass feedstock, ash formation, nonideal heat integration, incomplete reactant conversion, side product formation, and energy demand for auxiliaries (see Appendix A.6 for more details on the Aspen Plus models). Thus, this analysis assesses how much the process efficiency changes when considering nonidealities and whether the conclusions drawn with simpler models still stand.

The process efficiencies obtained with the detailed Power- and Biomass-to-Methanol models are 42 and 58% for the combustion-based and gasification-based processes, respectively. These values are a few percentage points lower than the ones calculated with the simpler models (ca. 46 and 70%). In particular, the efficiency of the detailed gasification-based process model deviates the most from the simpler one. Nevertheless, the gasification-based Power- and Biomass-to-Methanol process is significantly more efficient than the combustion-based one (58 vs 42%). Thus, the simpler process models were already able to capture the key inherent differences between the combustion-based and gasification-based processes.

The Sankey diagrams of the exergy flows in Figure 8 confirm that the efficiency difference between the combustion-based and gasification-based processes is mainly due to the lower exergy destruction in the electrolysis and biomass conversion units (see Figure 6 to compare the Sankey diagrams obtained with the simpler models).

The exergy efficiency of the methanol synthesis process (the “Synthesis” node in Figure 8) including product purification is ca. 85–90%, thus aligned with previous works^20^ and slightly lower than what is predicted with the simpler model. In contrast, the exergy efficiency of the gasifier is more noticeably lower than what was estimated with the simpler model (70 vs 85%) mainly due to the incomplete biomass conversion, side product formation, full oxidation of part of the carbon atoms (combustion reaction), and energy demand for syngas purification. The calculated efficiency of the gasifier is aligned with the work of Brown et al.^15^ The lower syngas quality and H_2_–C ratio due to side-product formation also require a higher hydrogen stream from the electrolysis unit, thus increasing the overall losses of the gasification-based Power- and Biomass-to-Methanol process compared to that of the simpler process model.

While the presented results considered methanol as product “X”, similar results were already obtained with detailed models of a combustion-based and gasification-based Power- and Biomass-to-Kerosene process,^19^ where the kerosene-like mixture can be approximated with the product dodecane, and are also expected for the other target products, i.e., methane and dimethyl ether. Thus, in summary, the considered simpler models allowed for identifying inherent differences for a wide range of products and feedstocks that were confirmed for two selected case studies with detailed models.

#### 5.3.5 Economics

In traditional combustion and gasification plants, air is often used as the oxidizing agent since oxygen has to be separated from the air in dedicated and expensive air separation units. In contrast, Power- and Biomass-to-X processes including water electrolysis already produce oxygen as a side product, which can be then used within the plant. Moreover, the possibility of using pure oxygen reduces the complexity, size, and cost of the plants since they can be smaller and additional separation units are not required thanks to the absence of the inert gas nitrogen.

Comparing the different pathways, we expect that gasification-based Power- and Biomass-to-X processes have lower costs than combustion-based Power- and Biomass-to-X processes. In fact, despite the higher costs for the biomass conversion unit^38^ and syngas cleaning, e.g., including the tar removal,^39^ no intermediate units like the RWGS unit are necessary (if most of the CO_2_ is separated and recycled into the gasifier^19^ or catalysts can convert both CO and CO_2_^40^) and significantly smaller power cycle and electrolysis units, which are often the major cost driver of Power-to-X processes, are required.

The economic benefits of gasification-based Power- and Biomass-to-X processes on both capital and operating expenditures can be seen for instance in Figure 9, where the economic results for the detailed model of the Power- and Biomass-to-Methanol (the technical results are shown in Section 5.3.4) are presented (refer to Section A.6 for details on the models and on the economic analysis). Despite the higher cost of the biomass conversion and flue gas purification units, the investment and operating costs for the electrolysis unit are significantly lower, thus making the gasification-based methanol production process more economically competitive (ca. 0.71 EUR/kg vs 1.07 EUR/kg). A similar trend was already shown for Power- and Biomass-to-Kerosene processes.^19^ Moreover, lower investment costs for storage of the reactants, e.g., oxygen and hydrogen, are needed in case of flexible operation of the whole gasification-based Power- and Biomass-to-X process.^41^

## 6 Conclusions

We investigated how residual biomass should be utilized in Power- and Biomass-to-X processes by modeling the main process units with mass and energy balances. In particular, two technologically mature thermochemical biomass conversion pathways, i.e., combustion and gasification, were evaluated and compared via an exergetic analysis by considering several products “X”, i.e., methane, methanol, dimethyl ether, and dodecane.

Gasification-based Power- and Biomass-to-X processes outperform the combustion-based ones in terms of efficiency for all the considered products (exergy efficiency around 15–20 percentage points higher), thus revealing that the carbon atoms should not be fully oxidized before the product synthesis. Therefore, the conversion of biomass to CO_2_ via combustion for the production of chemicals leads to suboptimal utilization of the biomass potential, which is instead valorized in gasification-based processes from an exergy perspective. There are two main reasons for this higher efficiency: First, the exergy loss is higher for the biomass combustion unit than for the gasification unit because the energy content of biomass is converted into heat rather than stored in the molecular bonds of the produced syngas. Second, the gasification pathway significantly reduces the amount of hydrogen produced via the electrolysis unit, which is the main contributor to exergy loss and investment costs in most of the Power-to-X processes. Moreover, gasification with steam instead of oxygen leads to higher efficiencies since syngas with a higher H_2_:CO ratio can be produced, thus reducing even further the amount of hydrogen that the electrolysis unit has to supply.

The sensitivity analyses conducted on electrolyzer efficiency, power cycle efficiency, and carbon feedstock composition show that the ranking does not change: gasification-based processes are more efficient than combustion-based ones. The electrolyzer efficiency has a strong influence on the efficiency of Power- and Biomass-to-X processes, differently from the efficiency of the power cycle converting the residual heat of reaction. Furthermore, the carbon feedstock composition that results in the highest efficiencies differs for the considered pathways.

Although the results clearly indicate the thermodynamic advantages of gasification-based Power- and Biomass-to-X processes, the calculated values only represent upper efficiency limits of real processes despite the use of empirical efficiency parameters for the electrolysis and power cycle units. In fact, the processes were modeled with simple mass and energy balances to identify inherent efficiency differences between the pathways. Thus, other aspects, e.g., nonideal reaction conversions, side reactions, energy demand for auxiliaries, and product separation, contribute to the reduction in the efficiency. For instance, the process efficiency was reduced from 46 to 42% and 70 to 58% for a combustion-based and gasification-based Power- and Biomass-to-Methanol process, respectively, when nonidealities were considered. Also, these nonidealities are expected to reduce the efficiency gap between combustion and gasification without changing the ranking, as shown for a Power- and Biomass-to-Methanol process. Furthermore, gasification-based Power- and Biomass-to-X processes might also be more promising from an economic perspective mainly due to the lower investment and operating costs of the electrolysis unit as shown for a Power- and Biomass-to-Methanol case study.

## Figures and Tables

**Figure A2:**
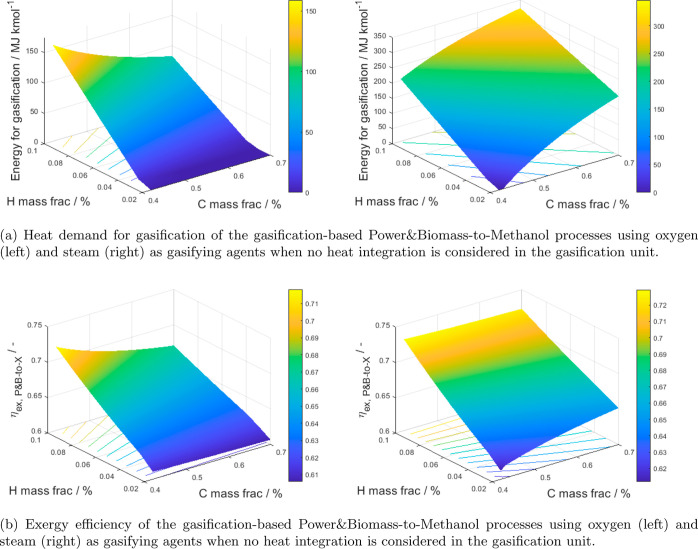
Sensitivity analysis of the heat demand for gasification of the Power- and Biomass-to-Methanol process with respect to the feedstock composition in the case heat integration within the gasification unit is not performed (a). The mass fractions of S and N were kept constant at 0.001 and 0.004, respectively (values for woody biomass); the mass fractions of C and H were varied, while the oxygen content was calculated by difference. When the energy demand for gasification is equal to 0, the gasification process is exothermic. The process exergy efficiency is shown in (b) to ease interpretation.

**Figure A3:**
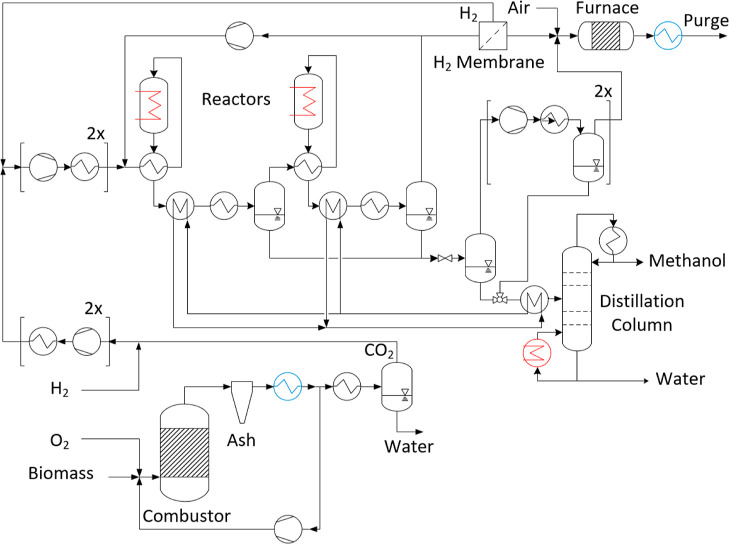
Simplified process flow diagram of the modeled combustion-based Power- and Biomass-to-Methanol process. Note: the electrolysis unit and the power cycle are not represented in the sketch since they were not modeled in Aspen Plus. Also, heat integration between the methanol synthesis reactors and the reboiler of the distillation column is not shown in the sketch for the sake of simplicity (the corresponding heat exchangers are marked in red). The heat exchangers in the biomass conversion and furnace units where heat is removed and supplied to the power cycle are marked in blue to ease identification.

**Figure A4:**
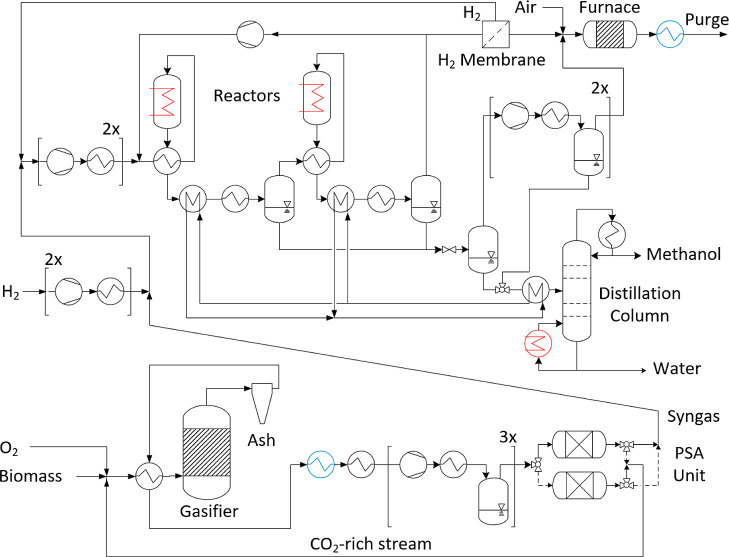
Simplified process flow diagram of the modeled gasification-based Power- and Biomass-to-Methanol process. Note: the electrolysis unit and the power cycle are not represented in the sketch since they were not modeled in Aspen Plus. Also, heat integration between the methanol synthesis reactors and the reboiler of the distillation column is not shown in the picture for the sake of simplicity (the corresponding heat exchangers are marked in red). The heat exchangers in the biomass conversion and furnace units where heat is removed and supplied to the power cycle are marked in blue to ease identification.

## A Appendix

### A.1 Molar balances

In Table A1, the reactions involved in each process unit are collected. As regards the synthesis units, multiple reactions are listed according to the inlet stream (CO_2_–H_2_ or CO–H_2_ mixture) and the target product, i.e., alkane (C_*n*_H_2*n*+2_) and alcohol or ether (C_*n*_H_2*n*+2_O).

**Table A1:** Complete Set of Reactions for the Considered Process Units

Combustion unit	
	A.1
Oxy-gasification unit	
	A.2
Steam-gasification unit	
	A.3
Electrolysis unit	
	A.4
Synthesis unit (CO_2_–H_2_ mixture)	
	A.5
	A.6
Synthesis unit (CO–H_2_ mixture)	
	A.7
	A.8

### A.2 Energy balances

A steady-state energy balance was written for each unit by neglecting the kinetic and potential energy contributions and heat losses. Except for the power cycle and the synthesis unit, no work exchange for either compression or expansion was considered.

The material streams at the interfaces of each unit are assumed at ambient conditions (*T*_0_ = 298 K, *P*_0_ = 1 bar). This choice implies the assumption of ideal heat integration between the inlet and outlet streams (Figure 4), thus making the whole reaction heat available at the typical reaction temperature of all the units except for the biomass combustion one. In the latter case, the reactants are heated up to the typical reaction temperature through the energy release of the exothermic reaction, while the product stream supplies heat to the power cycle, thus cooling down to the ambient temperature. This unit model better represents a real combustion unit and avoids overestimating the exergy flow (see Subsection A.4.1).

The assumption of perfect heat integration is acceptable for oxygen gasification since the streams have similar heat capacities and for steam gasification if an additional heat source is considered to generate steam, as done in this work. For water electrolysis instead, the heat capacity of the inlet and outlet streams differ significantly when considering ideal reaction conversion (no excess of water differently from what occurs in real applications). However, the energy needed to preheat the inlet stream is significantly lower (<1%) than the energy needed to drive the electrolysis; therefore, this energy demand was neglected. In contrast, for the synthesis unit, the change of phase of some products and byproducts during the cooling makes heat integration technically (and thermodynamically) challenging. This aspect has been considered in the exergy balance (see Subsection A.4.2).

Finally, no energy demand for product separation was considered. This assumption is acceptable when separating a gaseous product from water, while it is weaker for liquid products like methanol. Nevertheless, the separation process is analogous in the combustion- and gasification-based processes apart from the product concentration (higher in the gasification-based process). This means that these processes can still be compared to each other concerning the exergy efficiency, but the calculated values represent an upper limit.

### A.3 Modeling assumption overview

In Table A2, the key technical assumptions for the model of the Power- and Biomass-to-X units are summarized.

**Table A2:** Overview of the Key Technical Assumptions for the Model of the Power- and Biomass-to-X Units

unit	molar balance	energy balance
Combustion	- dry ash-free biomass feedstock	- no energy demand for biomass pretreatment
	- ideal reaction conversion (100%)	- operating temperature: 1000 °C
	- stoichiometric oxidant-to-fuel ratio	- cold product gas recycle for combustion temperature control
	- no side product formation	- combustion heat is provided to the power cycle
	- see Table A1 for reactions	- no heat losses
		- no energy demand for flue gas cleaning and CO_2_ separation
Gasification	- dry ash-free biomass feedstock	- no energy demand for biomass pretreatment
	- ideal reaction conversion (100%)	- operating temperature: 900 °C
	- stoichiometric oxidant-to-fuel ratio	- perfect heat integration between reactants and products
	- no side product formation	- if endothermic (typical operating condition), electric heating is considered
	- see Table A1 for reactions	- if exothermic, it is modeled as the combustion unit except for the operating temperature
		- no heat losses
		- no energy demand for flue gas cleaning and CO–H_2_ separation
Electrolysis	- ideal reaction conversion (100%)	- operating temperature: 60 °C
	- see Table A1 for reactions	- perfect heat integration between reactants and products
		- first-law efficiency: 60%
		- low-temperature heat is not recovered
Synthesis	- ideal reaction conversion (100%)	- product-dependent product synthesis pressure
	- stoichiometric feed stream	- energy demand for reactant compression is considered
	- no side product formation	- product-dependent product synthesis temperature
	- see Table A1 for reactions	- perfect heat integration between reactants and products
		- low-temperature heat from the synthesis reactor is provided to the power cycle
		- no energy demand for product separation
Power cycle		- power cycle efficiency for low-temperature heat: 30%
		- power cycle efficiency for high-temperature heat: 40%

### A.4 Exergy analysis

In the following, we provide additional information about how the exergy related to heat flows and material streams was calculated.

#### A.4.1 Exergy related to heat flows

The calculation of the exergy related to heat flows  was tailored to the models of the units. In the following, details on the exergy calculation are provided for each unit.

The exergy flow is calculated under the assumption of ideal gases as follows:

A.9

where *Q̇* is the available heat, and *T*_0_ and *T*_m_ are the ambient and thermodynamic mean temperatures.

For the combustion unit, the exhaust gases are cooled from the combustion temperature to the ambient temperature. Thus, *T*_m_ is equal to the logarithmic mean between these two temperatures. For the endothermic gasification unit, the exergy related to the heat transfer is assumed equal to the energy demand for gasification since it is assumed to be provided via electricity. Instead, for the exothermic gasification unit, the whole enthalpy of reaction is considered available and exchanged at the typical reaction temperature (*T*_m_). This implies the assumption of heat integration within the unit (Figure 4), as mentioned in Appendix A.2. The exergy related to the heat transfer of the electrolysis unit was calculated similarly to the exothermic gasification unit.

For the synthesis unit, the above-mentioned approximations would not be acceptable since the product stream changes phase partially or totally while cooling. Considering the whole enthalpy of reaction available at the reaction temperature would significantly overestimate the exergy value since the phase transition occurs at a temperature lower than the synthesis temperature. Therefore, the exergy associated with this heat transfer was calculated as the sum of three contributions, i.e., the exergy change for heating the reactants , the exergy change for cooling the products , and the exergy related to the heat transfer at the reaction temperature

A.10

 and  were calculated as the difference of the physical exergy at the ambient and synthesis temperatures, while  was calculated as in the gasification unit by considering the real amount of heat available at the reaction temperature. These data were obtained from the Aspen Plus database.

#### A.4.2 Molar Chemical Exergy

The chemical exergy of the material streams is calculated with respect to a reference environment.^42^ For the molecules in the reference environment, the molar chemical exergy for a pure component *k* (*e*_ch,*k*_) can be calculated with the following equation (numerical values in Table A3)

A.11

**Table A3:** Molar Chemical Exergy at 25 °C and 1 bar^42^

molecule	*e*_ch_/kJ mol^–1^
O_2_	4.0
CO_2_	20.1
H_2_O(l)	3.1

For other substances such as fuels, the molar chemical exergy value is often found in tables^42^ or calculated as a function of the LHV

A.12

As the molar chemical exergy values are known for all the considered gaseous and liquid fuels (Table A4), the factor ϕ was used only for biomass (assumed equal to 1.15, the lowest value in the range suggested by Kotas^42^).

**Table A4:** Considered Molar Chemical Exergy and LHV at 25 °C and 1 bar

molecule	*e*_ch_^a^/kJ mol^–1^	LHV^b^/MJ kg^–1^
H_2_	238.5	120.0
CO	275.4	10.1
CH_4_	836.5	50.0
CH_3_OH	722.6	19.9
CH_3_OCH_3_	1426.4	28.8
C_12_H_26_	8059.3	44.1

a Kotas.^42^

b Aspen Plus database.

The molar exergy of mixtures can be calculated as follows

A.13

However, the second term was neglected since it has a significantly lower value compared to the contribution of the sum of the molar exergy of the components (<1% for a 1:1 H_2_O–CH_3_OH mixture). Similarly, the minimum exergy demand for separation of the components of the mixture, which can be calculated via the abovementioned equation, was neglected.

### A.5 Additional Results of the Sensitivity Analyses

In this section, additional results concerning the conducted sensitivity analyses are presented.

#### A.5.1 Electrolyzer Efficiency

The effect of the efficiency of the electrolyzer on the overall Power- and Biomass-to-X efficiency (η_ex, P&B-to–X_) is shown in Table A5.

**Table A5:** Exergy Efficiency of the Power- and Biomass-to-X Processes with Biomass Combustion and Gasification for the Considered Products (Values in %)^a^

product	electrolyzer efficiency (%)	η_ex, P&B-to-X_ (combustion)	η_ex, P&B-to-X_ (O_2_-gasification)	η_ex, P&B-to-X_ (H_2_O-gasification)
CH_4_	60	43	59	61
CH_4_	80	54	70	71
CH_3_OH	60	46	68	72
CH_3_OH	80	56	78	79
CH_3_OCH_3_	60	46	69	72
CH_3_OCH_3_	80	57	78	79
C_12_H_26_	60	42	63	66
C_12_H_26_	80	52	72	73

a Sensitivity analysis with respect to the electrolyzer efficiency (based on the LHV). Woody biomass was considered as carbon feedstock.^21^

#### A.5.2 Power Cycle Efficiency

The results of the sensitivity analysis with respect to the efficiency of the power cycle unit are shown in Table A6. In particular, three cases are evaluated:

- The “30% and 20%” case, where 30% and 20% of the high and low-temperature heat are converted into electricity, respectively.
- The “40% and 30%” case (base case), where 40% and 30% of the high and low-temperature heat are converted into electricity, respectively.
- The “Carnot” case, where the maximum amount of electricity is produced from the residual heat without any exergy loss. A Carnot efficiency ranging from 47 to 56% is considered depending on the heat source temperature. Heat recovery from the electrolysis unit is also considered, differently from all the other cases. The corresponding Carnot efficiency is ca. 12%.

**Table A6:** Exergy Efficiency of the Power- and Biomass-to-X Processes with Biomass Combustion and Gasification for the Considered Products (Values in %)^a^

product	power cycle efficiency	η_ex, P&B-to-X_ (combustion)	η_ex, P&B-to-X_ (O_2_-gasification)	η_ex, P&B-to-X_ (H_2_O-gasification)
CH_4_	30% and 20%	42	58	60
CH_4_	40% and 30%	43	59	61
CH_4_	Carnot	48	63	65
CH_3_OH	30% and 20%	44	67	71
CH_3_OH	40% and 30%	46	68	72
CH_3_OH	Carnot	50	70	74
CH_3_OCH_3_	30% and 20%	44	68	71
CH_3_OCH_3_	40% and 30%	46	69	72
CH_3_OCH_3_	Carnot	50	72	75
C_12_H_26_	30% and 20%	41	62	65
C_12_H_26_	40% and 30%	42	63	66
C_12_H_26_	Carnot	47	66	69

a Sensitivity analysis with respect to the power cycle efficiency. Woody biomass was considered as carbon feedstock.^21^

#### A.5.3 Carbon Source Composition

Figure A1 shows the variation of the energy demand for gasification with respect to the feedstock composition. Steam-based gasification has a significantly higher energy demand for gasification, as already discussed in Subsection 3.1.3. Also, the exergy efficiency of the gasification-based Power- and Biomass-to-Methanol process is higher when the gasification process is endothermic, despite the higher energy input (recall Figure 7). However, the energy demand for real gasification processes might be higher; e.g., since real biomass feedstocks have some residual moisture, the oxidizing agent steam is generally supplied in excess, and perfect heat integration between the reactant and product streams is not possible. For instance, the energy demand for gasification increases significantly when assuming no heat integration within the gasification unit since the reactant stream has to be heated up to the reaction temperature (Figure A2). This additional energy demand reduces the efficiency of the gasification-based Power- and Biomass-to-X processes. Nevertheless, the efficiency is still higher than combustion-based processes. Moreover, the efficiency reduction between the gasification-based Power- and Biomass-to-X processes with and without heat integration would be even higher in the case of gasification with air as the gasifying agent due to the large amount of inert gases, e.g., nitrogen and argon, that has to be heated.

### A.6 Detailed Model of the Power- and Biomass-to-Methanol Processes

In the following, the Power- and Biomass-to-Methanol processes modeled in Aspen Plus are described. The thermodynamic models NRTL and PSRK were used for low-pressure and high-pressure process units, respectively.

#### A.6.1 Combustion-Based Power- and Biomass-to-Methanol Process

A woody biomass stream (see Table 1 for the composition) with 10 wt % moisture is considered as biogenic feedstock for the Power- and Biomass-to-Methanol process.

As shown in the simplified process flow diagram in Figure A3, biomass is preheated to ca. 175 °C by recycling part of the hot flue gases (ca. 85%) before being combusted with a stoichiometric amount of oxygen produced by the electrolyzer. The large flue gas recycle allows for maintaining the outlet temperature of the biomass oxy-combustor^43^ (modeled with an RGibbs reactor) at ca. 1000 °C. The choice of recycling flue gas rather than feeding excess oxygen (or air) eliminates the need for a carbon capture unit since the flue gas stream is mainly composed of H_2_O and CO_2_. Thus, a quite pure CO_2_ stream can be obtained from the flue gas via water condensation (see Figure A3). The other side products of the combustion unit (mainly ashes and SO_*x*_ if the combustion occurs at proper operating conditions) are considered to be fully removed in the flue gas treatment section. In particular, ashes are removed via a cyclone and a bag filter^44^ (a pressure loss of ca. 0.1 bar was accounted for), while the sulfur compounds are removed in situ, e.g., by using limestone in the fluidized bed and via wet scrubbing,^44^ which can achieve high removal efficiencies.^45^ These units were modeled in Aspen Plus with a separator block. The formation of NO_*x*_ was instead neglected since the concentration of N_2_ in the combustion chamber is low (oxy-combustion) and the combustion temperature is relatively low.^44^ Thus, the considered model of the flue gas cleaning treatment represents one of the most favorable cases. Additional flue gas cleaning steps as well as an additional energy demand would be needed if the impurity concentration exceeded the tolerance threshold of the catalyst of the downstream conversion process.

The heat from the flue gases with a temperature higher than 100 °C is recovered and converted into electricity in a power cycle with an efficiency of 40% (as in Subsection 3.4); in contrast, the residual heat that is removed to condense the water in the flue gases is considered wasted.

The CO_2_ stream from the biomass conversion unit is then mixed with a stoichiometric stream of green hydrogen produced in a water electrolyzer with an efficiency of 60% (as in Subsection 3.2). The CO_2_–H_2_ mixture is pressurized to 75 bar and preheated via heat integration before being supplied to the methanol synthesis section. Similarly to the process modeled in Mucci et al.,^46^ the methanol synthesis is modeled by using two water-cooled RPLUG reactors (with an LHHW kinetic model embedded^34,47^) operated at ca. 250 °C with intermediate removal of the product (crude methanol) from the unreacted gases via condensation in a flash unit (Figure A3). After the second methanol synthesis reactor, a second stream of crude methanol is separated from the unreacted gases, which are largely (98%) recycled.

Crude methanol is then purified via distillation to achieve a purity of ca. 99.5 wt %. The distillation unit (RADFRAC) has been sized to recover ca. 99.5% of the methanol in the feed stream. Heat for the reboiler of the distillation unit is supplied via heat integration with the methanol synthesis reactors. The remaining heat from the methanol synthesis reactors is recovered and converted into electricity in a power cycle with an efficiency of 30% (as in Subsection 3.4).

The high-pressure stream of unreacted gases is fed into a membrane unit (modeled via a shortcut model) to recover some hydrogen, which is recycled into the synthesis unit. In contrast, the low-pressure stream resulting from the methanol purification unit is compressed to recover additional methanol, which is supplied to the methanol purification unit (see Figure A3). These two gaseous streams are then combusted with air in a furnace, and the heat resulting heat is recovered and supplied to the power cycle (Figure A3).

#### A.6.2 Gasification-Based Power- and Biomass-to-Methanol Process

Analogously to the combustion-based Power- and Biomass-to-Methanol process, a woody biomass stream with 10 wt % moisture is considered as biogenic feedstock.

Oxygen from the electrolysis unit is the main gasifying agent for the biomass conversion unit. Nevertheless, a mixed oxygen-steam biomass gasifier is modeled with an RGibbs reactor since a significant amount of water is present in the biomass stream. In the gasification model, a biomass conversion factor of 95%^14^ is considered to account for incomplete biomass conversion due to the scarcity of the oxidizing agents. To reduce the heat demand for operating the gasifier at ca. 900 °C, the feed stream is preheated to ca. 450 °C via internal heat integration (see Figure A4). Similarly to the biomass gasification model in Mucci et al.,^19^ the gasifier is operated in an autothermal configuration,^14,31^ thus meaning that the heat demand for gasification is satisfied by supplying oxygen in excess compared to the stoichiometric amount (see Table A1): on the one hand, the need for an external high-temperature heat source is avoided; on the other hand, the combustion reaction is promoted and CO_2_ is produced. To improve the syngas quality, part of the CO_2_ is removed from the produced syngas via a Pressure Swing Adsorption unit simulated with a shortcut model based on Santos et al.^48^ (Figure A4). The separated CO_2_-rich stream is then recycled into the gasifier to decrease the carbon losses of the process and the need for a fresh gasifying agent. Similarly to the combustion unit model, the removal of the side products (ashes and sulfur-based compounds) is modeled by using a separator block. The formation of tars and the energy demand for their separation and cracking have not been included in the model since their concentration can be quite low when gasification occurs under proper operating conditions and in the presence of a catalyst. Thus, the modeled flue gas cleaning treatment of the gasification unit also represents one of the most favorable cases.

The H_2_–C ratio of syngas is then adjusted by adding green hydrogen produced in a water electrolyzer. The resulting stream is then pressurized and converted into methanol in a process designed similarly to the methanol synthesis unit described above for the combustion-based Power- and Biomass-to-Methanol process (see Figure A4).

Analogously to the model of the combustion-based Power- and Biomass-to-Methanol process, heat from the methanol synthesis reactors is supplied to the reboiler of the methanol purification unit. Furthermore, the residual heat from the methanol synthesis unit, the gasification unit, and the furnace is recovered and converted into electricity in a power cycle unit.

#### A.6.3 Economics

The levelized cost of methanol (*C*_MeOH_) for the modeled Power- and Biomass-to-Methanol processes was calculated as:

A.14

where CAPEX, O&M, and OPEX are the investment, operation and maintenance, and operating costs, respectively, *M*_MeOH, *y*_ is the yearly methanol production, *N* is the plant lifetime, and *i* is the interest rate. The replacement of the electrolysis unit (CAPEX_PEM_) in the 10th year is also considered.

The investment cost of the main equipment was estimated according to the correlations recommended by Biegler et al.^49^ More specific cost correlations were used for the biomass conversion unit,^38^ power cycle unit,^38^ water electrolysis unit (PEM technology),^50^ and methanol synthesis reactors.^51^ Utility and biomass prices in line with other literature works are considered to allow comparability, although electricity is often traded on overage at higher prices, e.g., in Germany. Additional assumptions of the economic analysis are collected in Table A7.

**Table A7:** Main Assumptions for the Economic Analysis

	values
Reference year	2021
USD to EUR conversion factor	0.85
plant lifetime	20 y
Yearly operating hours	8000
Yearly O&M costs	5% of the initial investment cost (CAPEX_0_)
Interest rate	5%
Electricity price	60 EUR/MWh
Cooling price	0.1 EUR/MWh
Biomass price	70 EUR/t

## Supporting Information Available

The molar, energy, and exergy flows of all the considered products and the efficiency of Power- and Biomass-to-X processes for real carbon feedstock compositions (PDF)
