# Supplementary material for: Combustion versus Gasification in Power- and Biomass-to-X Processes: An Exergetic Analysis
Source: ACS Omega. 2024 Nov 21;9(49):48213–31. doi: 10.1021/acsomega.4c05549 (PMC11635682; doi:10.1021/acsomega.4c05549)
Supplement: Supplementary file 1 — ao4c05549_si_001.pdf [file ao4c05549_si_001.pdf]

Combustion vs Gasification in Power- and Biomass-to-X Processes: An Exergetic Analysis Supporting Information

Simone Mucci[1,2], Alexander Mitsos[3,1,4], Dominik Bongartz[2,5,*]

[1] *Process Systems Engineering (AVT.SVT), RWTH Aachen University, 52074 Aachen, Germany*
[2] *Department of Chemical Engineering, KU Leuven, 3001 Leuven, Belgium*
[3] *JARA-ENERGY, 52056 Aachen, Germany*
[4] *Energy Systems Engineering (ICE-1), Forschungszentrum Jülich, 52425 Jülich, Germany*
[5] *EnergyVille, 3600 Genk, Belgium*
[*] *Corresponding author at: Department of Chemical Engineering, KU Leuven, 3001 Leuven, Belgium. E-mail address: dominikbongartz@alum.mit.edu*

1. Molar, energy, and exergy flows

Figures S1, S2, S3, and S4 show the molar flow rates for the considered Power- and Biomass-to-X processes when woody biomass is used as carbon feedstock, while Figures S5, S6, S7, and S8 the Sankey diagrams of the energy flows.

From the comparison of the molar flows for the considered processes, it can be noticed that a significantly lower amount of hydrogen is produced via electrolysis in the gasification-based processes. This leads to a lower electricity demand for the Power- and Biomass-to-X process and a higher energy efficiency per unit of product.

In Figures S5, S6, S7, and S8 can be noted that the electrolysis and power cycle units are the main contributors to the energy losses, meaning the energy that is not stored in the product, while the biomass conversion units have high energy efficiency in contrast to what was shown via the exergy analysis (see Section 5 of the main text). This result can be explained by the fact that the energy analysis does not consider the quality of the energy flows differently from the exergy analysis. In fact, the biomass conversion units significantly contribute to energy quality degradation (despite their high energy efficiency); in contrast, the power cycle units play a minor role in energy quality degradation since their energy input has already a relatively low quality.

The energy efficiency of the considered Power- and Biomass-to-X processes is shown in Figure S9. The ranking among the considered Power- and Biomass-to-X pathways with respect to the energy efficiency is analogous to the one shown for the exergy efficiency (see Figure 5 of the main text).

However, the energy and exergy efficiency values slightly differ due to the different ways of calculating the energy and exergy content of the products.

Finally, for the sake of completeness, the Sankey diagrams of the exergy flows for methane, dimethyl ether, and dodecane production are shown in Figures S10, S11, and S12.

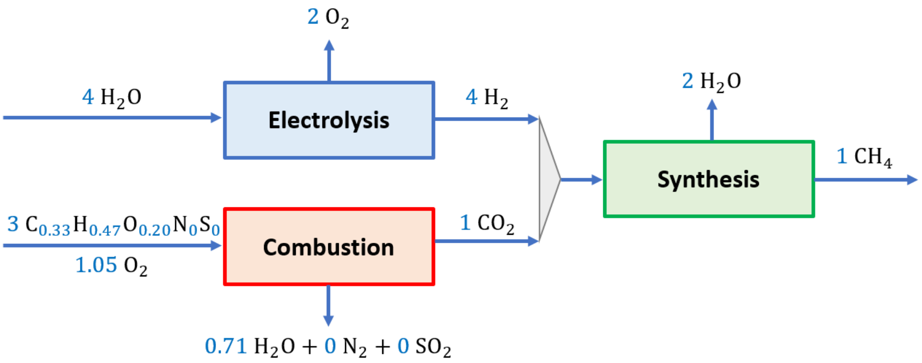

(a) Power- and Biomass-to-Methane process with biomass combustion.

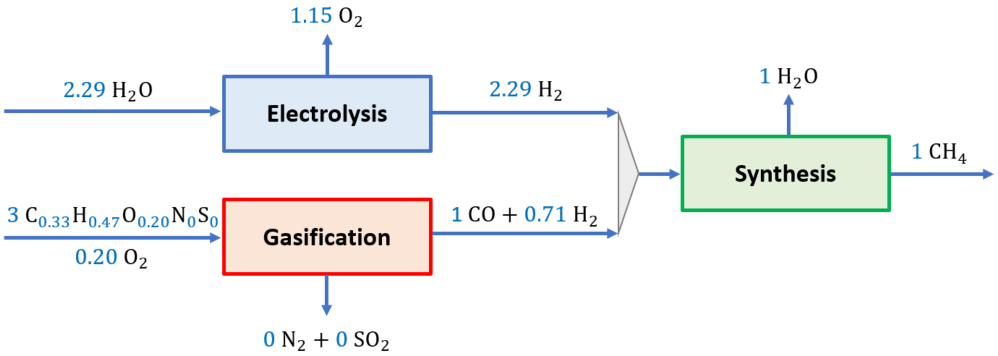

(b) Power- and Biomass-to-Methane process with biomass oxy-gasification.

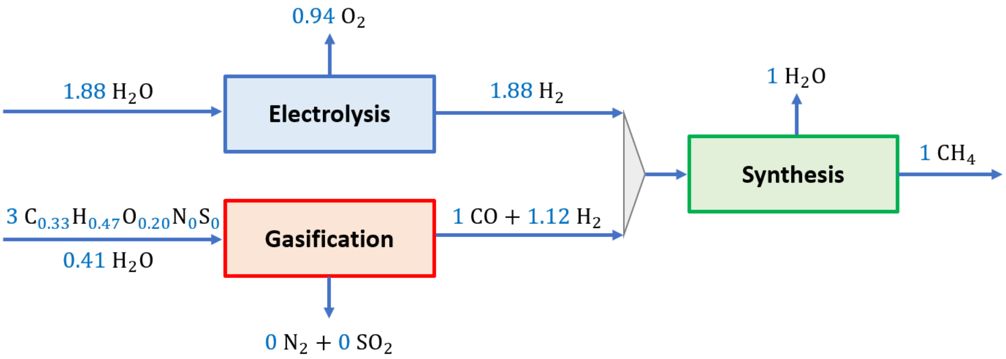

(c) Power- and Biomass-to-Methane process with biomass steam-gasification.

Figure S1: Molar flow rates in the Power- and Biomass-to-Methane process with (a) biomass combustion, (b) biomass oxy-gasification, and (c) biomass steam-gasification. The stoichiometric coefficients are shown in blue.

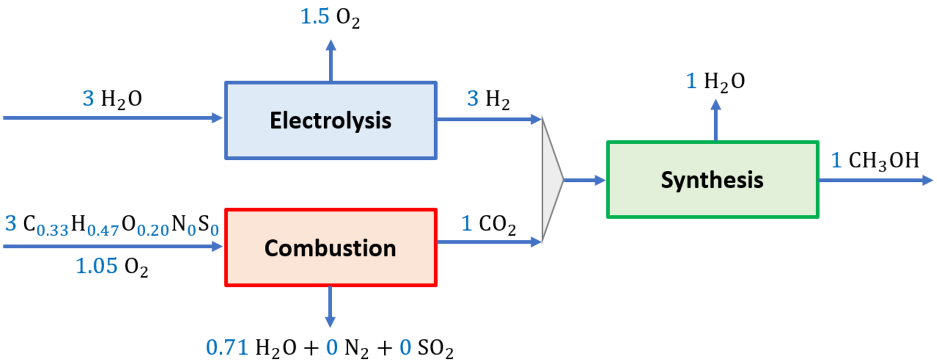

(a) Power- and Biomass-to-Methanol process with biomass combustion.

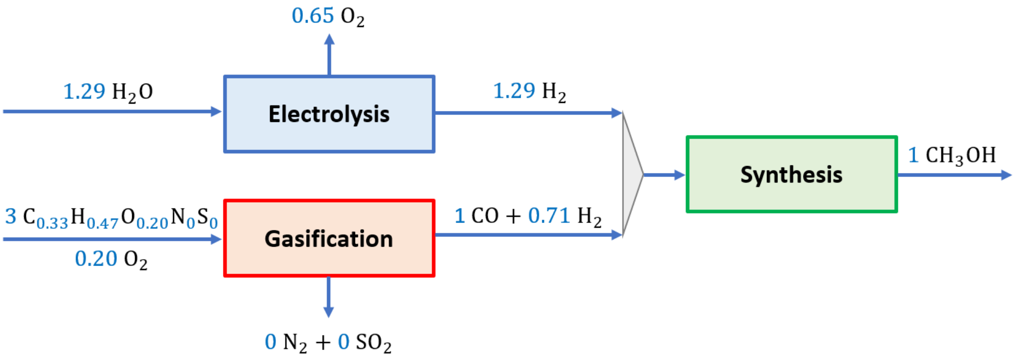

(b) Power- and Biomass-to-Methanol process with biomass oxy-gasification.

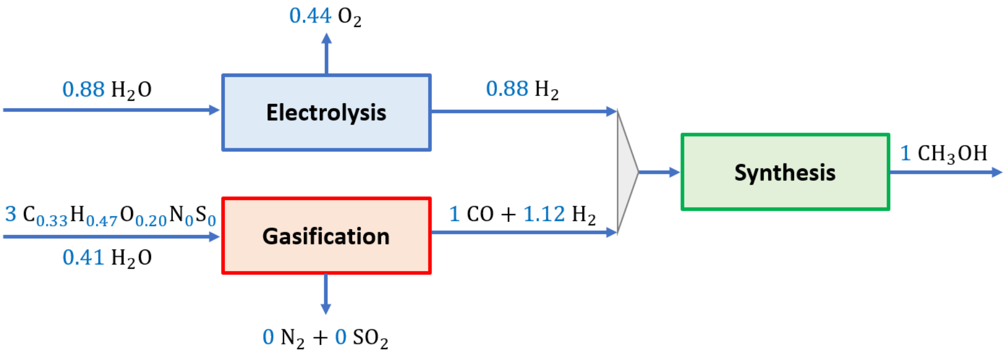

(c) Power- and Biomass-to-Methane process with biomass steam-gasification.

Figure S2: Molar flow rates in the Power- and Biomass-to-Methanol process with (a) biomass combustion, (b) biomass oxy-gasification, and (c) biomass steam-gasification. The stoichiometric coefficients are shown in blue.

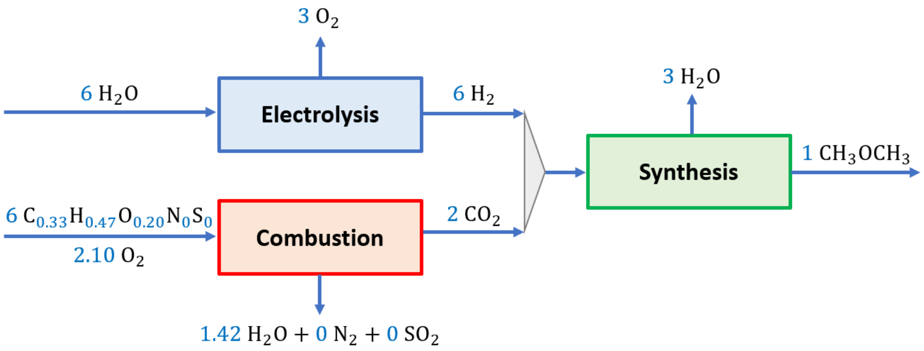

(a) Power- and Biomass-to-Dimethyl ether process with biomass combustion.

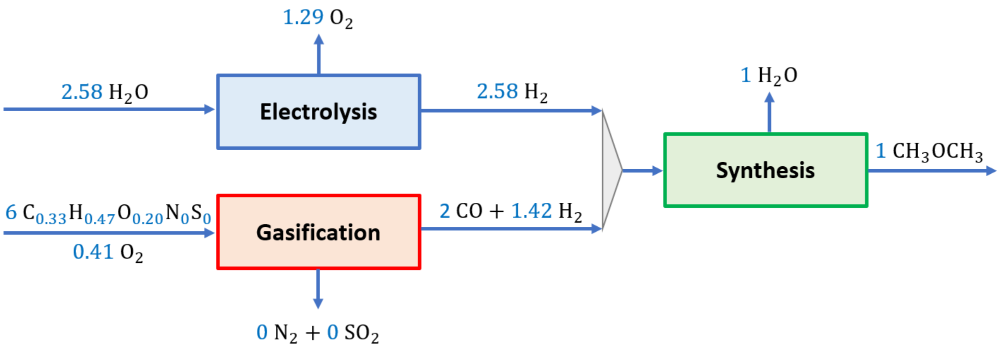

(b) Power- and Biomass-to-Dimethyl ether process with biomass oxy-gasification.

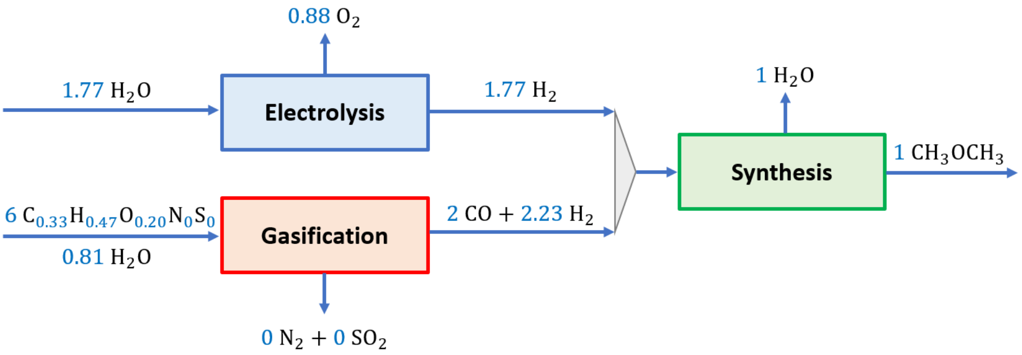

(c) Power- and Biomass-to-Dimethyl ether process with biomass steam-gasification.

Figure S3: Molar flow rates in the Power- and Biomass-to-Dimethyl ether process with (a) biomass combustion, (b) biomass oxy-gasification, and (c) biomass steam-gasification. The stoichiometric coefficients are shown in blue.

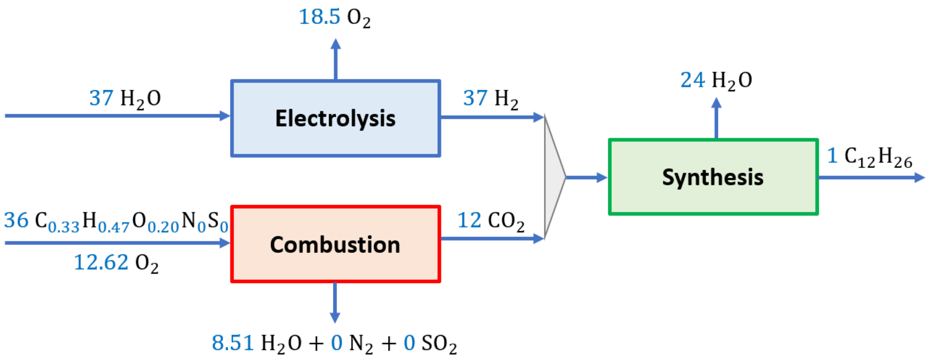

(a) Power- and Biomass-to-Dodecane ether process with biomass combustion.

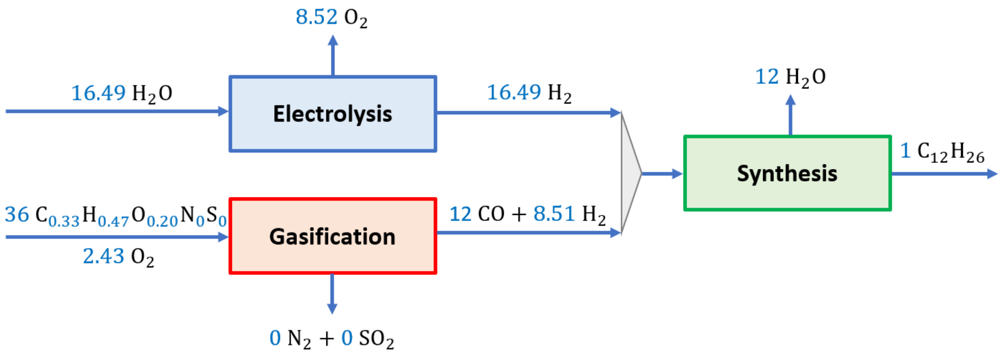

(b) Power- and Biomass-to-Dodecane ether process with biomass oxy-gasification.

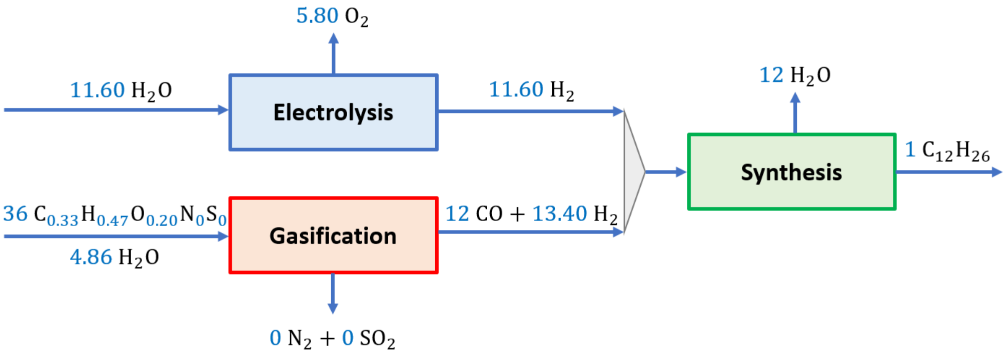

(c) Power- and Biomass-to-Dodecane ether process with biomass steam-gasification.

Figure S4: Molar flow rates in the Power- and Biomass-to-Dodecane process with (a) biomass combustion, (b) biomass oxy-gasification, and (c) biomass steam-gasification. The stoichiometric coefficients are shown in blue.

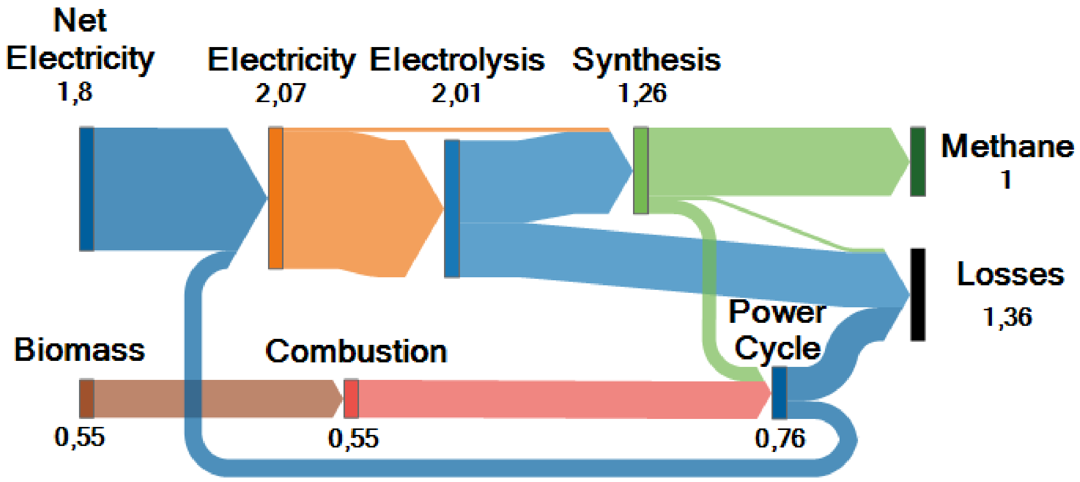

(a) Power- and Biomass-to-Methane process with biomass combustion.

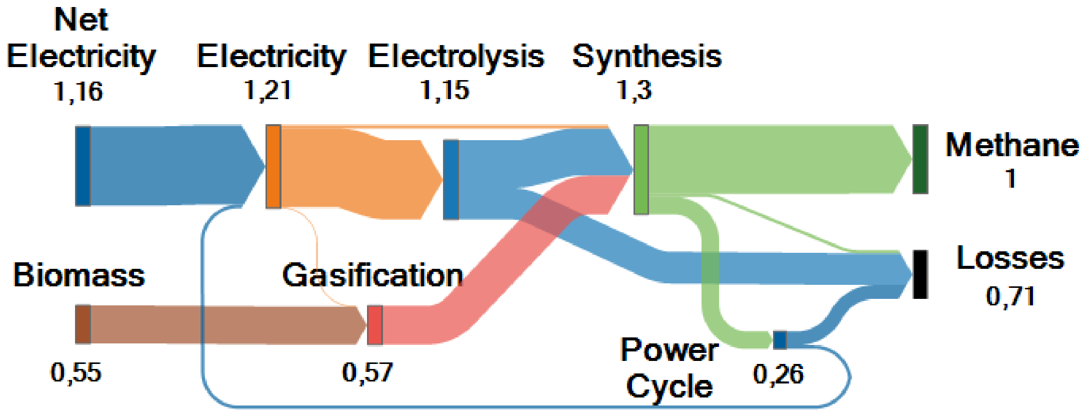

(b) Power- and Biomass-to-Methane process with biomass oxy-gasification.

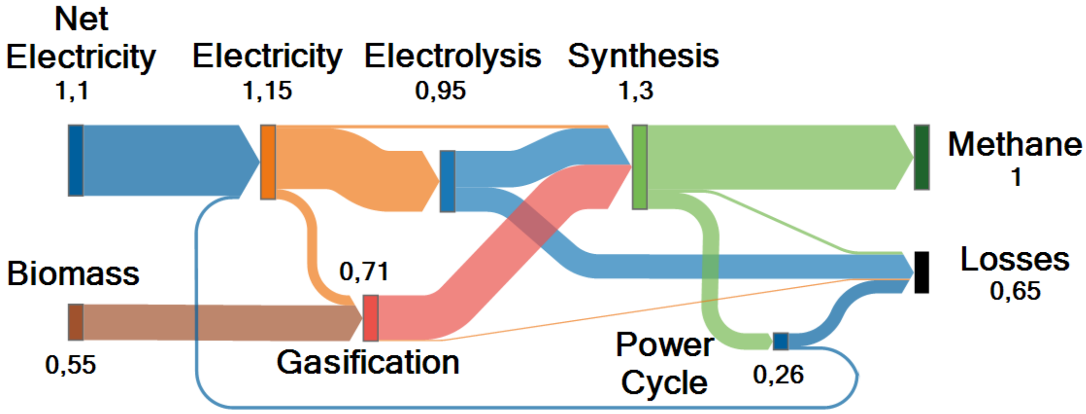

(c) Power- and Biomass-to-Methane process with biomass steam-gasification.

Figure S5: Sankey diagrams of the energy flows for the Power- and Biomass-to-Methane process with (a) biomass combustion, (b) biomass oxy-gasification, and (c) biomass steam-gasification. The energy flows in MW are scaled per unit of product and are based on the LHV of the components. The numbers at the diagram nodes represent the energy flows of the energy sources and the total energy flows through the process units.

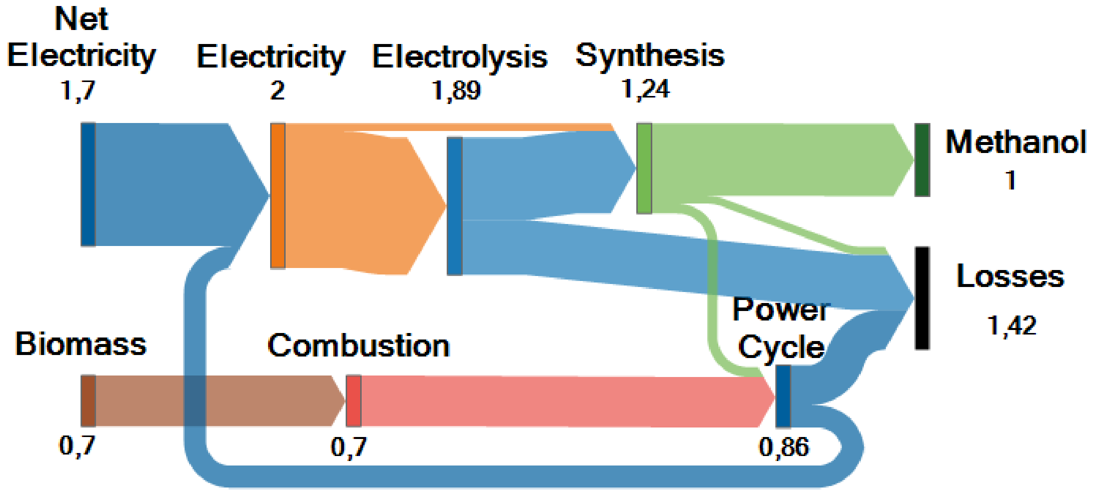

(a) Power- and Biomass-to-Methanol process with biomass combustion.

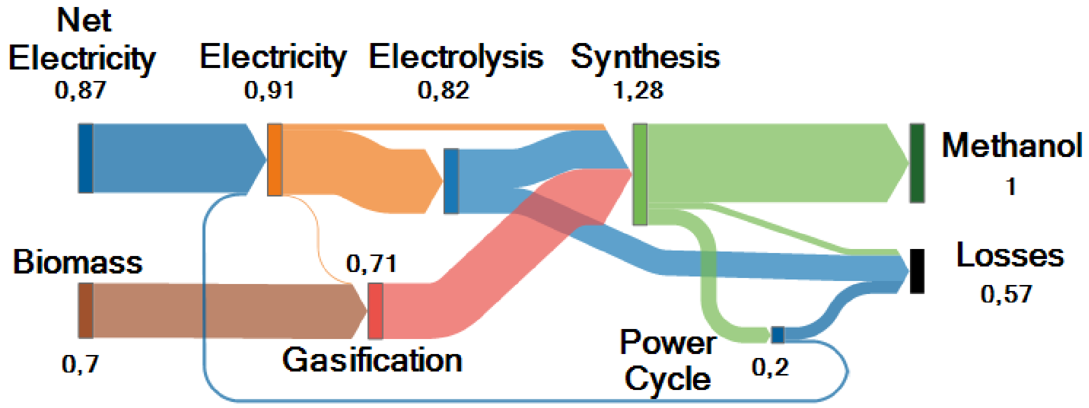

(b) Power- and Biomass-to-Methanol process with biomass oxy-gasification.

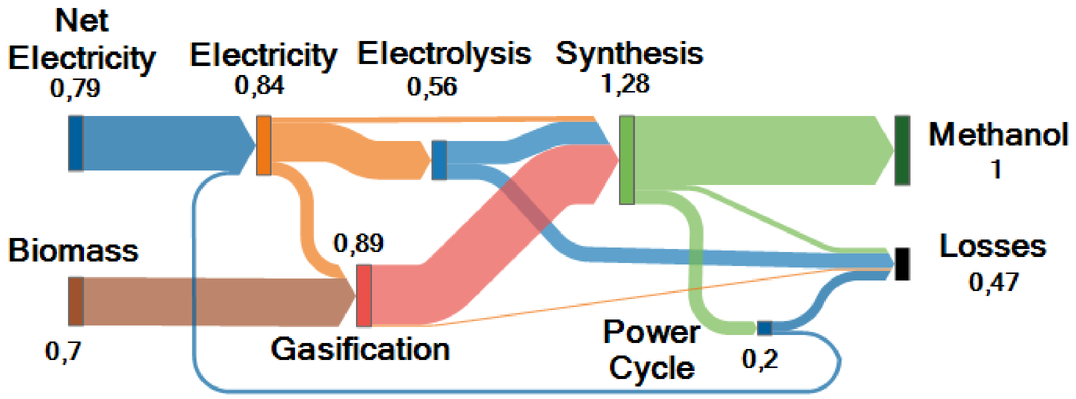

(c) Power- and Biomass-to-Methanol process with biomass steam-gasification.

Figure S6: Sankey diagrams of the energy flows for the Power- and Biomass-to-Methanol process with (a) biomass combustion, (b) biomass oxy-gasification, and (c) biomass steam-gasification. The energy flows in MW are scaled per unit of product and are based on the LHV of the components. The numbers at the diagram nodes represent the energy flows of the energy sources and the total energy flows through the process units.

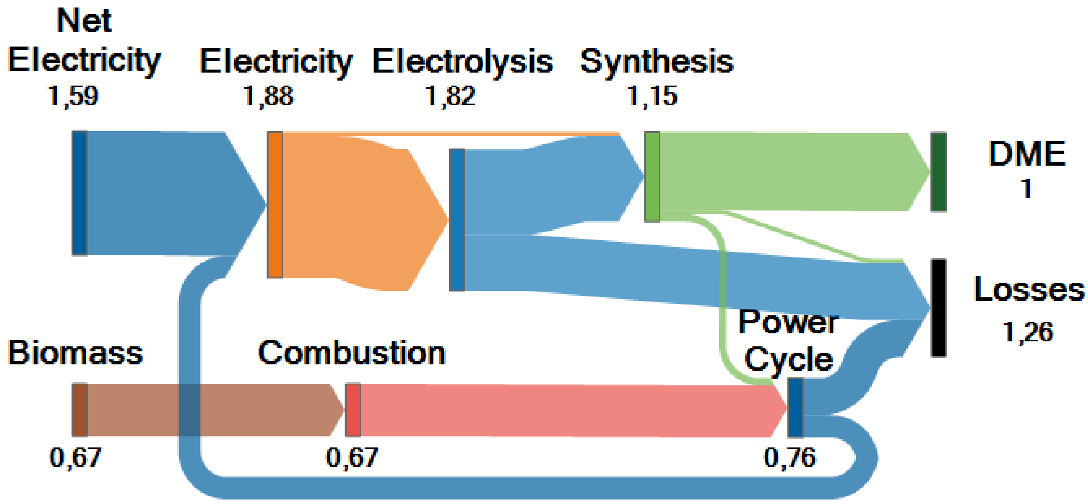

(a) Power- and Biomass-to-Dimethyl ether process with biomass combustion.

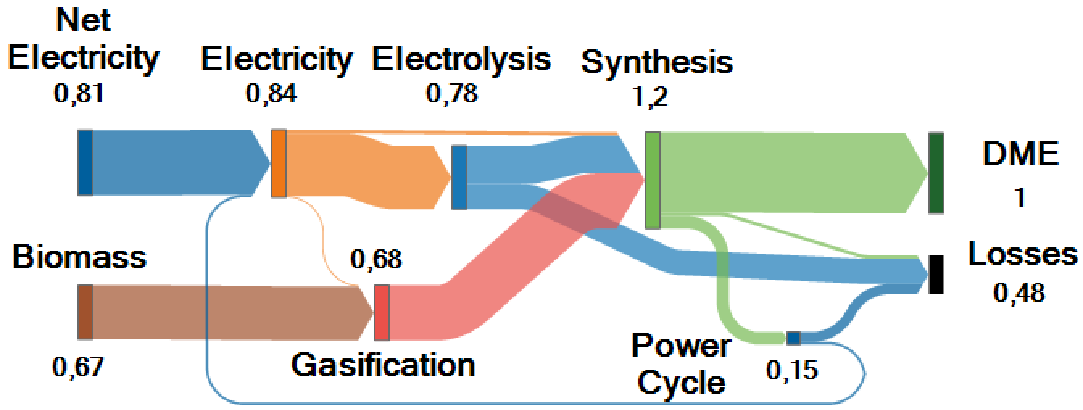

(b) Power- and Biomass-to-Dimethyl ether process with biomass oxy-gasification.

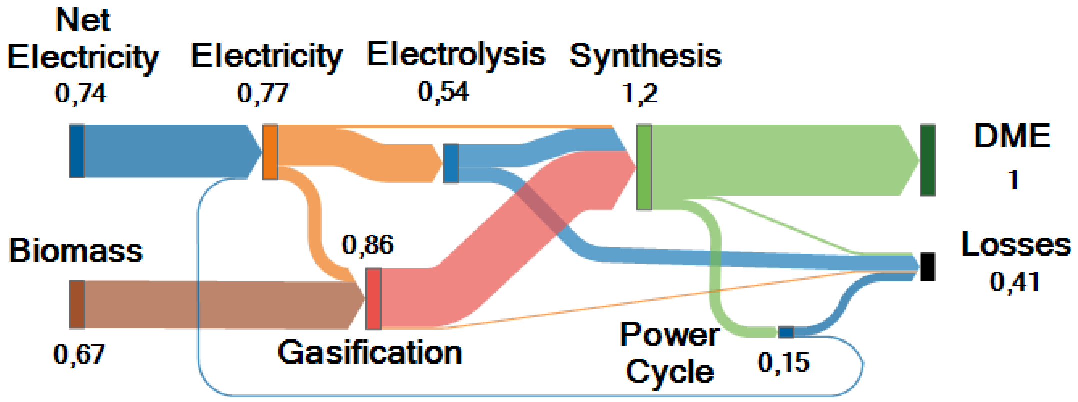

(c) Power- and Biomass-to-Dimethyl ether process with biomass steam-gasification.

Figure S7: Sankey diagrams of the energy flows for the Power- and Biomass-to-Dimethyl ether (DME) process with (a) biomass combustion, (b) biomass oxy-gasification, and (c) biomass steam-gasification. The energy flows in MW are scaled per unit of product and are based on the LHV of the components. The numbers at the diagram nodes represent the energy flows of the energy sources and the total energy flows through the process units.

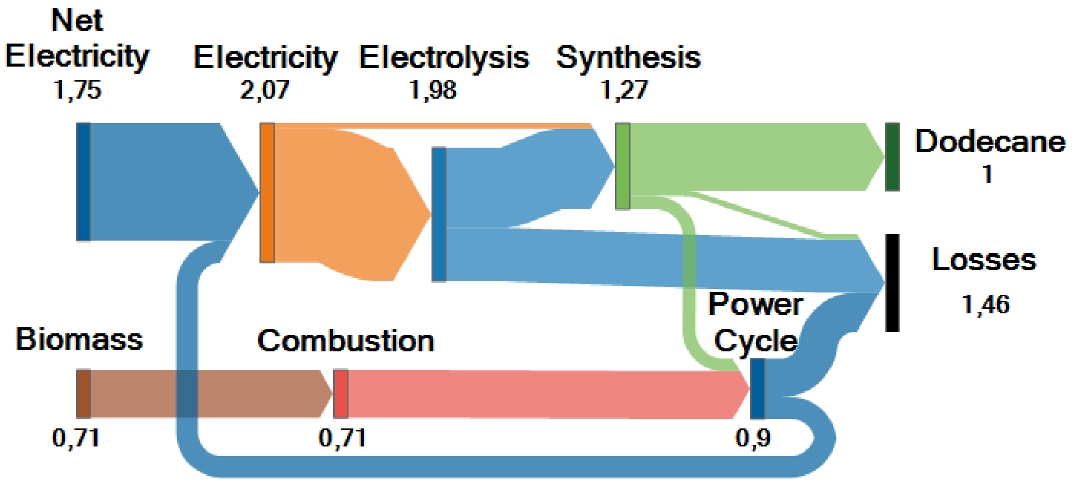

(a) Power- and Biomass-to-Dodecane process with biomass combustion.

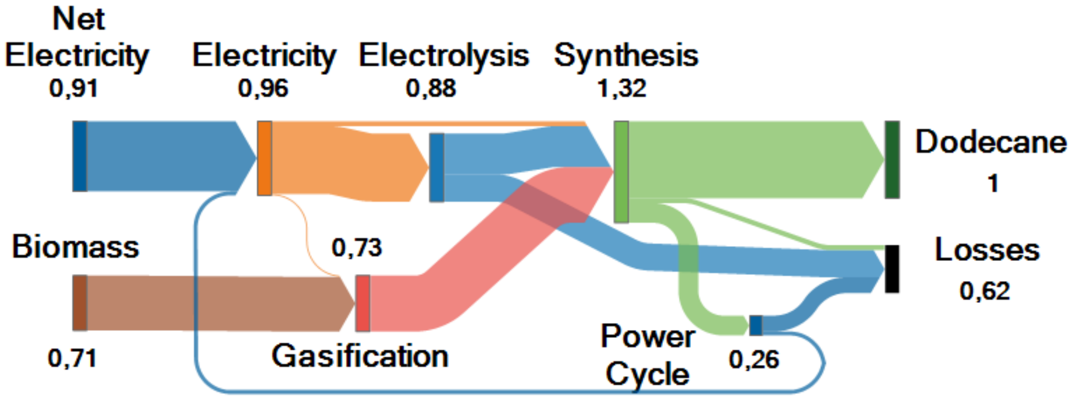

(b) Power- and Biomass-to-Dodecane process with biomass oxy-gasification.

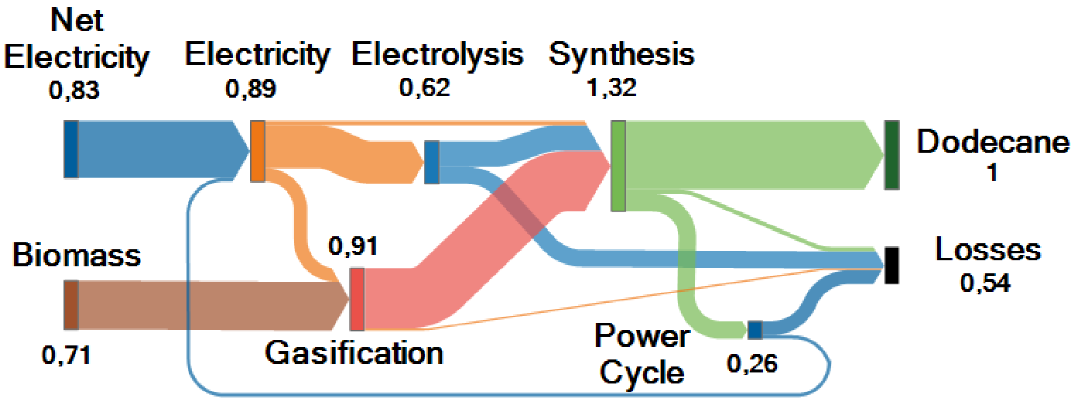

(c) Power- and Biomass-to-Dodecane process with biomass steam-gasification.

Figure S8: Sankey diagrams of the energy flows for the Power- and Biomass-to-Dodecane process with (a) biomass combustion, (b) biomass oxy-gasification, and (c) biomass steam-gasification. The energy flows in MW are scaled per unit of product and are based on the LHV of the components. The numbers at the diagram nodes represent the energy flows of the energy sources and the total energy flows through the process units.

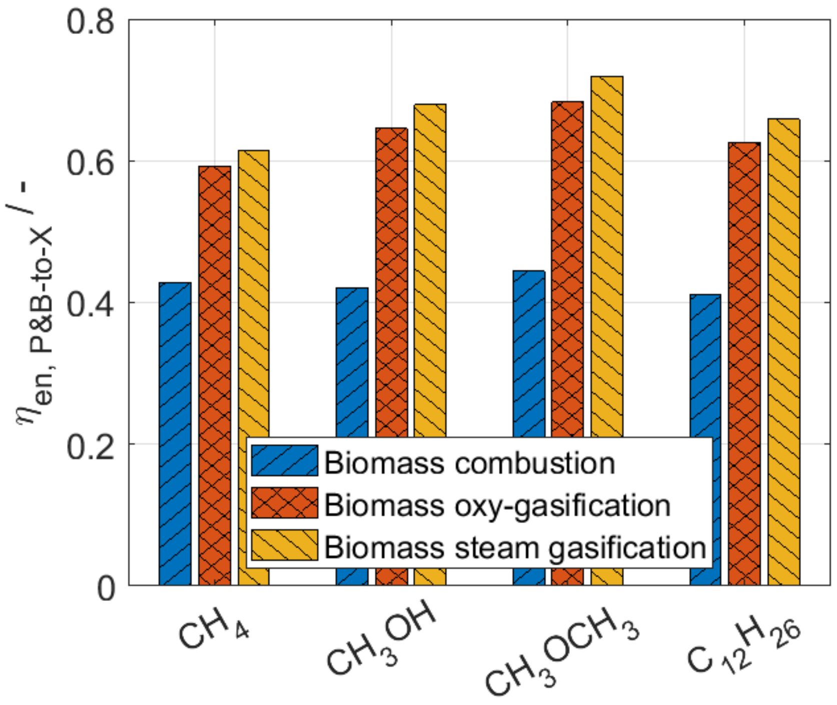

Figure S9: Energy efficiency of Power- and Biomass-to-X processes with biomass combustion and gasification for different products ‘X’. Woody biomass is considered as carbon feedstock [1].

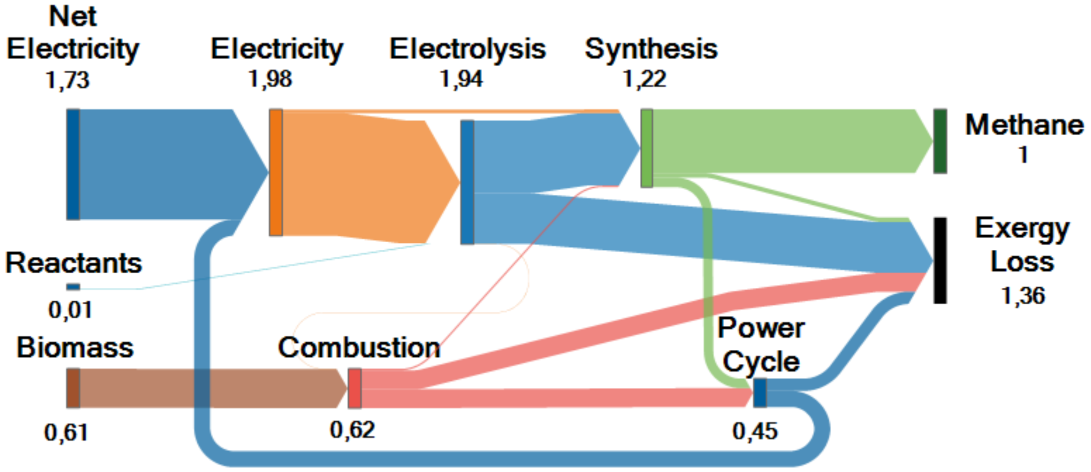

(a) Power- and Biomass-to-Methane process with biomass combustion.

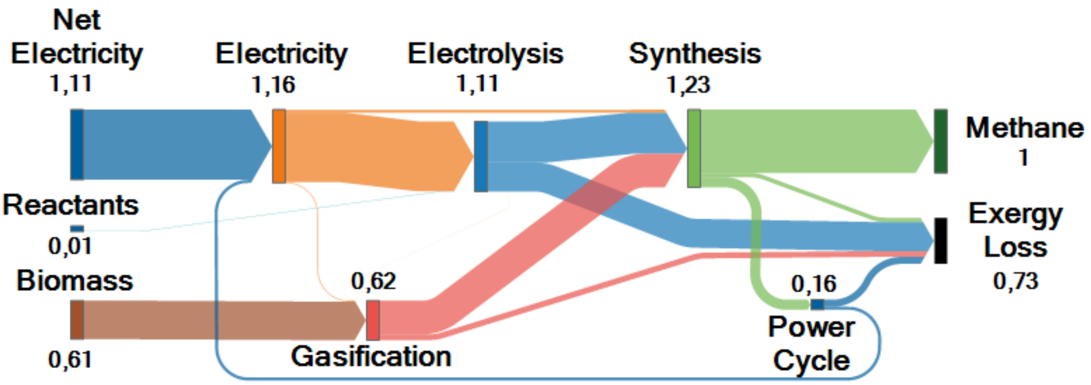

(b) Power- and Biomass-to-Methane process with biomass oxy-gasification.

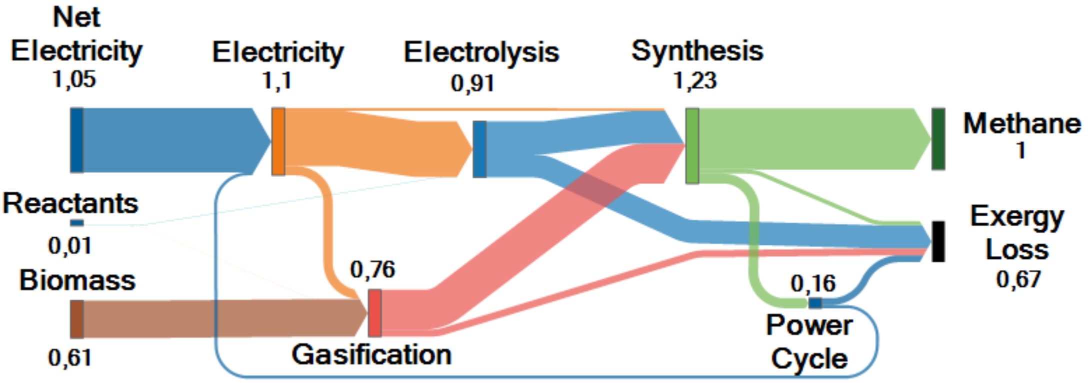

(c) Power- and Biomass-to-Methane process with biomass steam-gasification.

Figure S10: Sankey diagrams of the exergy flows for the Power- and Biomass-to-Methane process with (a) biomass combustion, (b) biomass oxy-gasification, and (c) biomass steam-gasification. The exergy flows in MW are scaled per unit of product. The numbers at the diagram nodes represent the exergy flows of the energy sources and the total exergy flows through the process units.

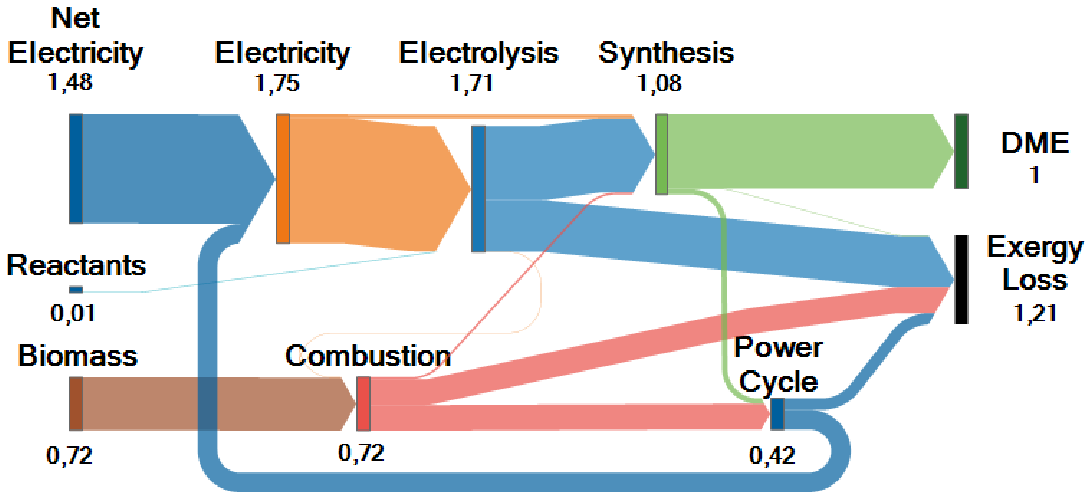

(a) Power- and Biomass-to-Dimethyl ether process with biomass combustion.

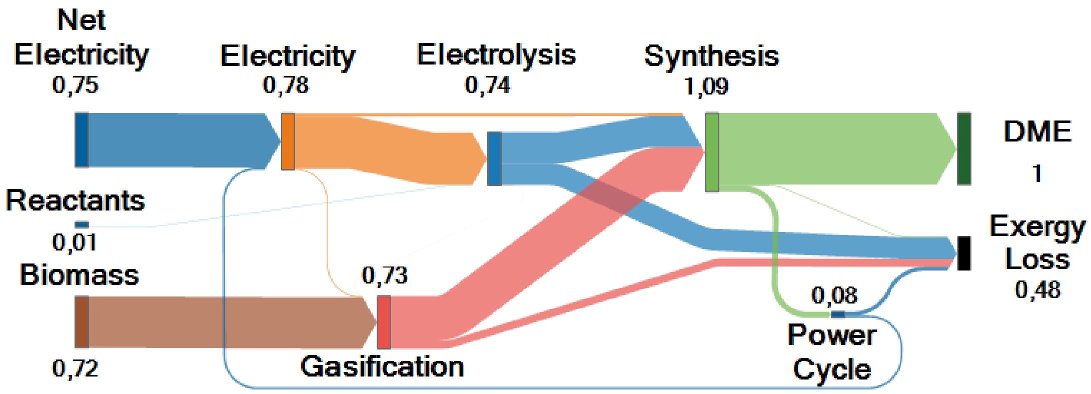

(b) Power- and Biomass-to-Dimethyl ether process with biomass oxy-gasification.

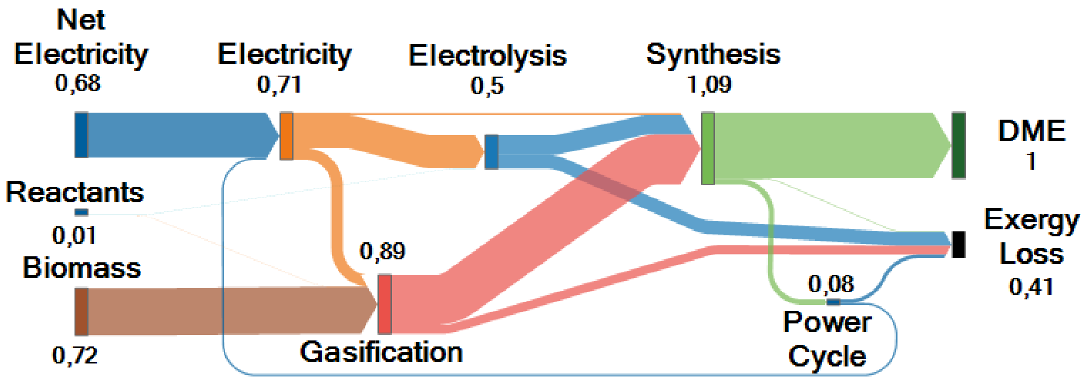

(c) Power- and Biomass-to-Dimethyl ether process with biomass steam-gasification.

Figure S11: Sankey diagrams of the exergy flows for the Power- and Biomass-to-Dimethyl ether (DME) process with (a) biomass combustion, (b) biomass oxy-gasification, and (c) biomass steam-gasification. The exergy flows in MW are scaled per unit of product. The numbers at the diagram nodes represent the exergy flows of the energy sources and the total exergy flows through the process units.

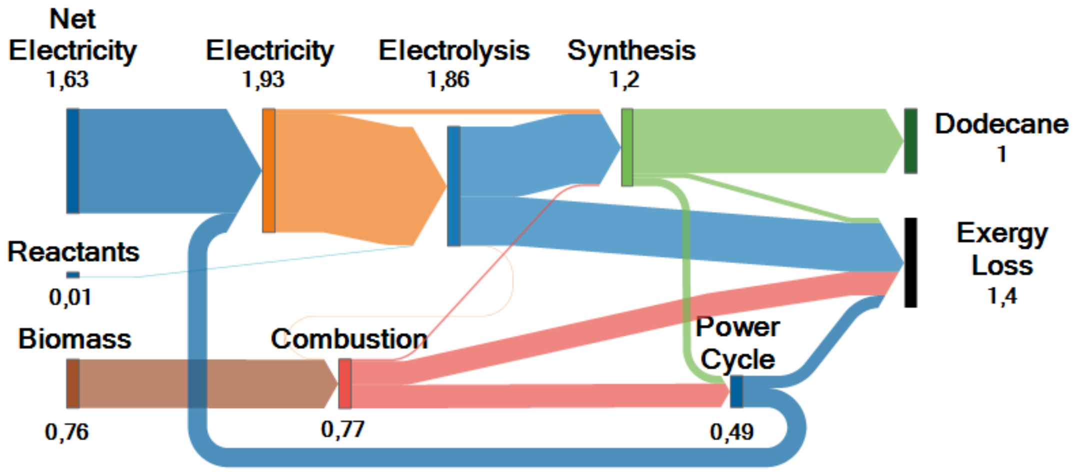

(a) Power- and Biomass-to-Dodecane process with biomass combustion.

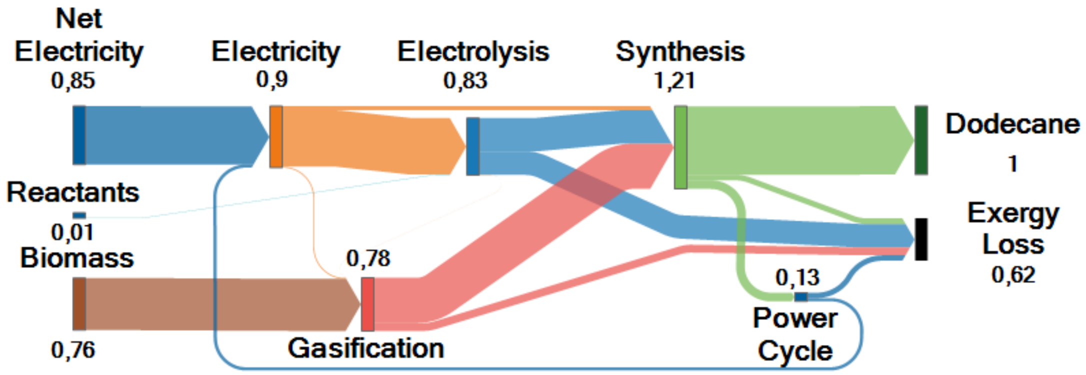

(b) Power- and Biomass-to-Dodecane process with biomass oxy-gasification.

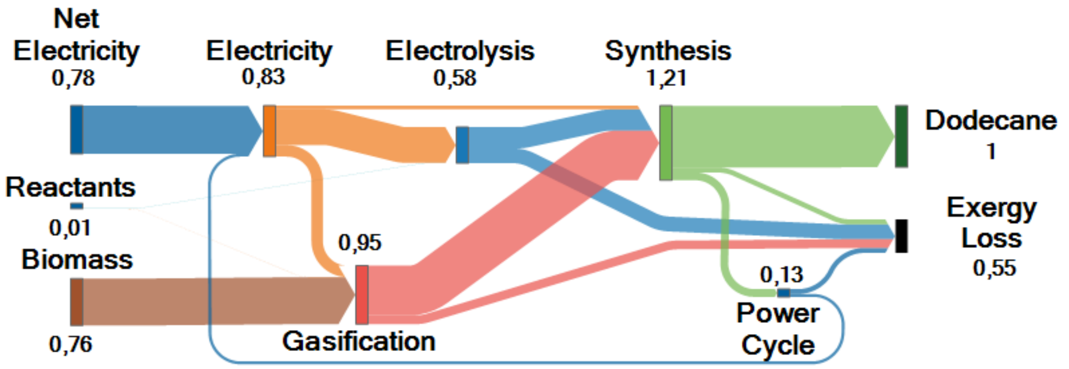

(c) Power- and Biomass-to-Dodecane process with biomass steam-gasification.

Figure S12: Sankey diagrams of the exergy flows for the Power- and Biomass-to-Dodecane process with (a) biomass combustion, (b) biomass oxy-gasification, and (c) biomass steam-gasification. The exergy flows in MW are scaled per unit of product. The numbers at the diagram nodes represent the exergy flows of the energy sources and the total exergy flows through the process units.

2. Power- and Biomass-to-X efficiency for real carbon feedstock compositions

In this section, the exergy efficiencies of the Power- and Biomass-to-X processes have been calculated under the same assumptions of the paper for several carbon feedstocks, ranging from biomass and biogas to municipal solid waste (Table S1). Ash-free and dry carbon feedstocks were considered as input (see Table S2 for the composition).

The lower heating value (LHV) of the feedstock was calculated according to the correlations proposed by Sheng and Azevedo [2] and Boumanchar et al. [3] for the biomass-related and waste-related feedstocks, respectively. The LHV of biogas was instead calculated by assuming a molar composition of 60 % CH_4 and 40 % CO_2. Finally, the considered ϕ factors for the chemical exergy calculation (see Appendix A.4.2 in the main text) were 1.15 and 1.02 for biomass-related and biogas feedstocks, respectively [4]. The ϕ factor of waste-related feedstock was assumed equal to the ϕ factor of biomass (1.15).

As already observed in the sensitivity analysis of the carbon feedstock (see Section 5.2.3 in the main text), the exergy efficiency of gasification-based processes is more affected by the carbon feedstock composition than the exergy efficiency of combustion-based processes. Also, the effect of the feedstock variation on the process efficiency is higher when the H:C ratio of the product is low. Nevertheless, gasification-based Power- and Biomass-to-X processes are always more efficient under the model assumptions, irrespective of the feedstock composition.

Among the considered feedstocks, biogas results to be the best alternative. This is mainly due to the fact that the ϕ factor is significantly lower than biomass. However, the direct comparison with the other feedstocks is not completely fair since biogas is a derivative of biomass via anaerobic digestion for which the conversion process was not included in the efficiency calculation. Nevertheless, its use would also have advantages in real plants since the pre-processing of the biogenic feedstock avoids ashes and pollutants in its conversion process to valuable chemicals: biogas combustion or reforming instead of biomass combustion or gasification can be used to produce either CO_2 or syngas, thus reducing the effort for solid handling in the conversion process.

Apart from biogas, marine macroalgae are the most promising biomass-related feedstock for gasification with respect to the calculated exergy efficiencies. However, the high concentration of sulfur might be problematic in real applications. Paper waste and plastics and rubber waste are instead the most efficient waste-related feedstocks for oxygen-based and steam-based gasification. However, the gasification of waste, especially plastic waste, is less mature [5].

Finally, it is important to highlight that other practical aspects such as the feedstock moisture, sulfur and ash contents (not considered in this analysis), can make these feedstocks not suitable for direct use in Power- and Biomass-to-X processes. Moreover, an energy-intense carbon feedstock pre-treatment and flue gas post-treatment might be needed, which can significantly affect the overall process efficiency.

Table S1: Exergy efficiency of the Power- and Biomass-to-X processes with biomass combustion and gasification for the considered products (values in %). Sensitivity analysis with respect to the feedstock composition.

**Product**	**Feedstock**	$\eta_{\text{ex, P\&B-to-X}}$ (combustion)	$\eta_{\text{ex, P\&B-to-X}}$ (O_2-gasification)	$\eta_{\text{ex, P\&B-to-X}}$ (H_2O-gasification)
CH_4	Woody biomass	43	59	61
CH_4	Herbaceous and agricultural biomass	43	59	61
CH_4	Mixture of biomass	43	59	61
CH_4	Marine macroalgae	43	60	61
CH_4	Biogas	44 #	65 *#	66 *#
CH_4	Paper	44	60	60
CH_4	Plastics and rubber	42	57	63
CH_4	Solid waste incinerator	44	§	§
CH_3OH	Woody biomass	46	68	72
CH_3OH	Herbaceous and agricultural biomass	45	69	72
CH_3OH	Mixture of biomass	46	68	72
CH_3OH	Marine macroalgae	45	70	72
CH_3OH	Biogas	47	79 *	81 *
CH_3OH	Paper	46	70	71
CH_3OH	Plastics and rubber	44	66	75
CH_3OH	Solid waste incinerator	47	§	§
CH_3OCH_3	Woody biomass	46	69	72
CH_3OCH_3	Herbaceous and agricultural biomass	46	69	72
CH_3OCH_3	Mixture of biomass	46	68	72
CH_3OCH_3	Marine macroalgae	45	70	72
CH_3OCH_3	Biogas	47	79 *	81 *
CH_3OCH_3	Paper	46	70	71
CH_3OCH_3	Plastics and rubber	44	66	76
CH_3OCH_3	Solid waste incinerator	47	§	§
$C_{12}H_{26}$	Woody biomass	42	63	66
$C_{12}H_{26}$	Herbaceous and agricultural biomass	42	63	66
$C_{12}H_{26}$	Mixture of biomass	42	63	66
$C_{12}H_{26}$	Marine macroalgae	42	64	66
$C_{12}H_{26}$	Biogas	44	73 *	74 *
$C_{12}H_{26}$	Paper	43	64	65
$C_{12}H_{26}$	Plastics and rubber	41	61	69
$C_{12}H_{26}$	Solid waste incinerator	44	§	§

# Biogas upgrading would be more efficient for the production of methane.
* Biogas is reformed instead of gasified.
§ Too high oxygen content for the considered model of gasification

Table S2: Ash-free dry composition of the carbon feedstock in %wt. (ultimate analysis).

	x_C	x_O	x_H	x_N	x_S	**Source**
Woody biomass (mean)	52.1	41.2	6.2	0.4	0.1*	[1]
Herbaceous and agricultural biomass (mean)	49.9	42.6	6.2	1.2	0.1*	[1]
Mixture of biomass (mean)	52.9	39.6	6.2	1.0	0.3*	[1]
Marine macroalgae	43.2	45.8	6.2	2.2	2.6	[1]
Biogas (60-40 %vol. CH_4-CO_2)	43.2	48.0	8.8	0	0	
Paper (mean)	43.4	50.2	5.9	0.4	0.1*	[3]
Plastics and rubber (mean)	71.9	17.7	9.2	0.8	0.4*	[3]
Solid waste incinerator (mean)	29.7	65.5	3.8	0.7	0.3*	[3]

* Adapted/Rounded

References

[1] S. V. Vassilev, D. Baxter, L. K. Andersen, and C. G. Vassileva. An overview of the chemical composition of biomass. *Fuel*, 89(5):913–933, 2010. doi:10.1016/j.fuel.2009.10.022.

[2] C. Sheng and J. L. Azevedo. Estimating the higher heating value of biomass fuels from basic analysis data. *Biomass and Bioenergy*, 28(5):499–507, 2005. doi:10.1016/j.biombioe.2004.11.008.

[3] I. Boumanchar, Y. Chhiti, F. E. M. Alaoui, A. Sahibed-dine, F. Bentiss, C. Jama, and M. Bensitel. Municipal solid waste higher heating value prediction from ultimate analysis using multiple regression and genetic programming techniques. *Waste Management and Research*, 37:578–589, 2019. doi:10.1177/0734242X18816797.

[4] T. J. Kotas. *The Exergy Method of Thermal Plant Analysis.* Krieger Publishing Company, 1995.

[5] G. Lopez, M. Artetxe, M. Amutio, J. Alvarez, J. Bilbao, and M. Olazar. Recent advances in the gasification of waste plastics. A critical overview. *Renewable and Sustainable Energy Reviews*, 82:576–596, 2018. doi:10.1016/j.rser.2017.09.032.
